# More than a simple epithelial layer: multifunctional role of echinoderm coelomic epithelium

**DOI:** 10.1007/s00441-022-03678-x

**Published:** 2022-09-09

**Authors:** Silvia Guatelli, Cinzia Ferrario, Francesco Bonasoro, Sandra I. Anjo, Bruno Manadas, Maria Daniela Candia Carnevali, Ana Varela Coelho, Michela Sugni

**Affiliations:** 1 Department of Environmental Science and Policy, University of Milan, Via Celoria 26, 20133 Milan, Italy; 2 Center for Complexity & Biosystems, Department of Physics, University of Milan, Via Celoria 16, 20133 Milan, Italy; 3 CNC-Center for Neuroscience and Cell Biology, University of Coimbra, Rua Larga, 3004-504 Coimbra, Portugal; 4 ITQB-Instituto de Tecnologia Química e Biológica António Xavier, Universidade Nova de Lisboa, Av. da República, 2780-157 Oeiras, Portugal

**Keywords:** Echinoderms, Coelomic Epithelium, Proteome, Dedifferentiation, Immune response

## Abstract

In echinoderms, the coelomic epithelium (CE) is reportedly the source of new circulating cells (coelomocytes) as well as the provider of molecular factors such as immunity-related molecules. However, its overall functions have been scarcely studied in detail. In this work, we used an integrated approach based on both microscopy (light and electron) and proteomic analyses to investigate the arm CE in the starfish *Marthasterias glacialis* during different physiological conditions (i.e., non-regenerating and/or regenerating). Our results show that CE cells share both ultrastructural and proteomic features with circulating coelomocytes (echinoderm immune cells). Additionally, microscopy and proteomic analyses indicate that CE cells are actively involved in protein synthesis and processing, and membrane trafficking processes such as phagocytosis (particularly of myocytes) and massive secretion phenomena. The latter might provide molecules (e.g., immune factors) and fluids for proper arm growth/regrowth. No stem cell marker was identified and no pre-existing stem cell was observed within the CE. Rather, during regeneration, CE cells undergo dedifferentiation and epithelial-mesenchymal transition to deliver progenitor cells for tissue replacement. Overall, our work underlines that echinoderm CE is not a “simple epithelial lining” and that instead it plays multiple functions which span from immunity-related roles as well as being a source of regeneration-competent cells for arm growth/regrowth.

**Supplementary Information:**

The online version contains supplementary material available at 10.1007/s00441-022-03678-x.

## Introduction

Coelomic epithelium (CE), or coelothelium, is a mesodermal-derived tissue lining coelomic cavities and enveloping inner organs in all coelomates, including vertebrates, where it forms both peritoneum and pericardium, as well as pleura, in mammals.

Due to its histogenetic potential, CE is considered a key tissue characterized by unique features. Indeed, during vertebrate embryonic development, it is involved in organogenesis of the heart, gut, lungs, liver, and gonads (Ariza et al. 2016) as well as in morphogenesis of other complex structures, such as limb bud (Gros and Tabin 2014), providing fundamental signals necessary for their development. In adults, it is involved in body fluid homeostasis and transport, in cooperation with immune system cells, and in cytokine and growth factor synthesis (Mutsaers and Wilkosz 2007; Ariza et al. 2016). Besides vertebrates, CE is well known to play a significant role in all invertebrate coelomates. In echinoderms, it was regarded as “ancient multifunctional organogenetic tissue” (Candia Carnevali et al. 1995b; Candia Carnevali and Bonasoro 2001; Dolmatov et al. 2007). Indeed, several studies demonstrated its importance during both embryonic and post-embryonic (larval or adult) development in various echinoderm species (Candia Carnevali et al. 1995a; Candia Carnevali et al. 2009; Hernroth et al. 2010). For example, in both visceral regeneration in holothurians and arm regeneration in “stellate” echinoderms, CE gives an indispensable contribution to the development of somatic or visceral muscles (Candia Carnevali et al. 1995a; Dolmatov et al. 2007; García-Arrarás and Dolmatov 2010; Ben Khadra et al. 2018). This is in line with the remarkable development, diversification, and complexity of coelom and coelom-derived structures in adult echinoderms, which have no equal among the coelomates. Indeed, the CE lines the perivisceral coelom, the echinoderm-exclusive water vascular system (e.g., the radial water canals, the inner side of ampullae, and tube feet), and the peri-haemal system (Hyman 1955), all these compartments displaying a multiplicity of functions.

At present, in adult echinoderms, CE is supposed to play fundamental roles acting as follows:

1. *Source of stem/multipotent cells* (undifferentiated coelomocytes), particularly during regenerative phenomena (Candia Carnevali et al. 2009; Candia Carnevali and Burighel 2010; Hernroth et al. 2010; García-Arrarás et al. 2011). Echinoderms possess striking capabilities to regenerate new functional tissues, whole organs, or extensive body parts after their loss or injury (Candia Carnevali 2006; García-Arrarás et al. 2011; Ben Khadra et al. 2018; Ferrario et al. 2018). Several recent studies report that during the regenerative events, widespread centers of cell cycle activity are detected in the epithelium of the different coelomic compartments (somatocoelic, hydrocoelic, axocoelic) (Dolmatov 1993; Candia Carnevali and Bonasoro 2001; Biressi et al. 2010; Hernroth et al. 2010; Ben Khadra et al. 2017). In holothuroids, following muscle injuries, CE cells de-differentiate and migrate into the damaged zone contributing to the regeneration of longitudinal muscles (Ginanova 2007). In addition, the CE is also fundamental for gut regeneration in the sea cucumber *Holothuria glaberrima*, where coelothelial cells undergo epithelial-mesenchymal transition (EMT) and colonize the underlining connective tissue, eventually participating to the reconstruction of the digestive tract (García-Arrarás et al. 2011).
2. *Direct source of circulating cells* (differentiated coelomocytes) including immune cells, which are indispensable elements during the first repair events of clotting and defense against pathogens (Bossche and Jangoux 1976; Muñoz-Chápuli et al. 2005; Pinsino et al. 2007). At least part of these cells can be considered the functional (and in some cases morphological, Andrade et al 2021) analogs of vertebrate blood cells (e.g., thrombocytes, macrophages), although they differ in the origin and a much wider morphofunctional complexity/diversity is described for the latter. However, it is important to note that in echinoderms, the term “coelomocytes” is used to refer to heterogeneous cell populations which can differ from class to class in terms of both morphology and function such as presumptive stem cells as well as immune cells (Smith et al. 2010; Andrade et al. 2021). Present knowledge on the origin of coelomocytes is still incomplete and controversial, and the presumptive coelomocyte-producing tissue has not been definitively identified. Although many structures such as the axial organ (Golconda et al. 2019), Tiedemann’s bodies (Kaneshiro and Karp 1980), and recently the pharynx (Golconda et al. 2019) have been regarded as hypothetical primary sources of coelomocytes, current research considers the CE in its entirety the most probable candidate for this role. In particular, different authors observed a remarkable morphological and functional similarity between cells released from the CE and circulating coelomocytes (Kozlova et al. 2006; Holm et al. 2008; Sharlaimova and Petukhova 2012).Notably, in the starfish *Asterias rubens*, a few hours after arm amputation, local CE proliferation is followed by an increased number of circulating coelomocytes (Pinsino et al. 2007). Gorshkov and colleagues (2009) observed a release of immature flagellated cells from the CE and hypothesized that those cells could be the precursors of coelomocytes. More recently, in the same species, Sharlaimova et al. (2014, 2021) identified a sub-population of small undifferentiated cells in the CE context and suggested these elements as putative progenitors and source of new circulating coelomocytes. This hypothesis was also supported by proteomic analyses highlighting partially shared protein profiles between the CE and the circulating coelomocytes (Sharlaimova et al. 2021).

Despite these indications, the histological approach alone, microscopic or ultrastructural, is not sufficient to fully attribute a coelomocyte-producing role to the CE and even the presumptive production/release process of stem/pluripotent cells from coelothelium during regeneration needs to be confirmed as well. As a matter of fact, it is still unclear if the intense proliferation detectable in regenerating CE is functional to the production of wandering coelomocytes necessary to restore the post-traumatic fluid loss or rather to produce truly regeneration-competent cells used as building blocks of the regenerating tissues. In order to overcome some of these issues and deeply understand the complex physiological role of this epithelial tissue in echinoderm biology, it is important to integrate morphological evidences with detailed molecular data.

In particular, proteomics is becoming a powerful tool to deepen the problem of cell and tissue functions during both tissue homeostasis and regeneration by depicting the existing protein activities involved in the regrowth processes. However, proteomics investigations focused on echinoderm CE are quite limited. Specific molecular information is due to the works by Gabre et al. (2015) and by Kim et al. (2018), which are focused on the characterization of the CE transcriptome from the clonal starfish *Coscinasterias muricata* and the blue bat starfish *Patiria pectinifera*, respectively. The analysis of the biological processes associated with the transcripts identified in these studies suggests the involvement of CE in the development of anatomical units. More recently, the CE proteome of the common starfish *Asterias rubens* was also published (Sharlaimova et al. 2021). Despite these evidences, there is a great lack of integrated multidisciplinary studies that can contribute to unravel the complex roles exerted by this tissue both in regeneration and in other physiological processes.

In order to partially unravel this gap, the present work is addressed to analyze the CE of the starfish *Marthasterias glacialis* in both standard physiological (homeostatic) and regenerating conditions, by combining histological/ultrastructural analyses with appropriate proteomics approaches, the former to provide a detailed cell and tissue perspective, the latter to contribute a baseline molecular view of CE multifunctional implications. This integrated approach can significantly help to shed new light on the intriguing role of this apparently simple but fundamental tissue.

## Materials and methods

### Experimental animals and maintenance

Adult specimens of *Marthasterias glacialis* with a size ranging from 15 to 35 cm were collected at low tide on the west coast of Portugal (Estoril, Cascais). The animals were transferred to the “Vasco da Gama” Aquarium (Dafundo, Oeiras) where they were left to acclimatize in open-circuit tanks with re-circulating sea water at 15 °C and 33‰ salinity. They were fed ad libitum with mussels collected at the same site.

Twelve animals were used for regeneration tests and ultrastructural analyses, whereas six non-regenerating starfish were used for proteomic analyses.

### Regeneration tests

Starfish were anesthetized with 4% (w/v) MgCl_2_ in sea water and two arm tips (1–2 cm from the arm tip) for each animal were removed with a scalpel. After amputation, the animals were put back into the aquarium and left to regenerate. Selected regenerating time-points were 24 h, 48 h, and 1 week post-amputation (p.a.). For each time point, three animals were used and at the end of the regenerating period, both regenerating arm tips (including 0.5–1 cm of the stump) were removed from each animal. Additionally, two arm tips were collected from each of three non-regenerating individuals. All the samples were immediately fixed in a solution of 2% glutaraldehyde and 1.2% NaCl in 0.1 M sodium cacodylate at 4 °C for at least 4 h and then processed for ultrastructural analyses.

### Ultrastructural analyses

Samples were processed using the transmission electron microscopy (TEM) protocol described in Ferrario et al. (2018). Briefly, both regenerating and non-regenerating arm tips were washed in 0.1 M sodium cacodylate buffer, post-fixed in 1% osmium tetroxide in the same buffer for 2 h, washed in distilled water, and left in a decalcifying solution (1:1 (v/v) mixture of 2% ascorbic acid in dH_2_O and 0.3 M NaCl) for 24 h. Samples were washed in dH_2_O and left in 2% uranyl acetate in 25% ethanol (EtOH) for 2 h, dehydrated in an EtOH series (25–100%), cleared in propylene oxide, washed in Epon 812-Araldite:propylene oxide solutions in different proportions (1:3, 1:1, 3:1), and finally embedded in pure Epon 812-Araldite.

Semithin (about 1 µm) and ultrathin (70–90 nm) sections were performed using a Reichert-Jung Ultracut E. Semithin sections were stained with crystal violet and basic fuchsin and photographed under a Jenaval light microscope provided with a Leica EC3 Camera and Leica Application Suite LAS EZ Software (Version 1.8.0). Ultrathin sections were mounted on copper grids and stained with 1% uranyl acetate followed by lead citrate. They were then observed and photographed using a CM10 Philips TEM equipped with a Morada Soft Imaging System digital camera with Item software or EFTEM Leo912ab (Zeiss).

### Coelomic epithelium proteome characterization

Proteomic analyses were carried out on the CE of non-regenerating animals. Two arms were removed from each starfish with a scalpel performing a cut at their base, close to the central disk. Each arm was opened and the perivisceral CE was removed from the inner oral and aboral sides of the arm with tweezers. The CE collected from different animals were pooled together to obtain three independent biological replicates and immediately frozen in liquid nitrogen.

### Total protein extraction

Water-soluble protein extracts for each replicate were obtained after an automated frozen tissue disruption procedure as described by Butt and Coorssen (2006). Briefly, the deep-frozen CE was placed in a previously chilled Teflon capsule containing 3 stainless steel beads (5 mm in diameter). The capsule was placed in a MikroDismembrator (Sartorius) and set to 3000 rpm for 2.5 min. According to Righetti’s protocol (Fasoli et al. 2011), the deep-frozen powdered sample was re-suspended in PBS buffer (20 mM phosphate-buffered saline, pH 7.2, containing 5 mM ethylenediaminetetraacetic acid (EDTA) and 1 mM dithiothreitol), supplemented with a protease inhibitor cocktail 0.2× (SigmaFast, Sigma). The homogenate was ultra-centrifuged at 36000×*g* for 10 min at 4 °C using the Optima TLX Ultracentrifuge (4.N7/12) with the TLS-55 rotor (Beckman-Coulter) to separate the insoluble debris. After the separation, both the supernatant containing the total protein extract and the pellet were frozen at −80 °C until use. The total protein concentration of each replicate was determined using the QuantiPro BCA Assay Kit according with the manufacturer’s protocol. Before quantification, the protein extracts were diluted 1:300 to reduce the interference of DDT and EDTA.

### Tryptic digestion of total protein extracts

For each replicate, the volume of protein extracts corresponding to 100 µg of protein was adjusted to 20 µL using 6 M urea in 50 mM ammonium bicarbonate (AB). Two micrograms of recombinant green fluorescent protein (MBP-GFP) was added to each protein sample as an internal standard for retention time adjustment and intensity normalization. The tryptic digestion was performed following a previously published protocol (Gomes et al. 2019). It started with the reduction of protein disulfide bonds, followed by their alkylation before the incubation with trypsin. Vacuum-dried (Scientific Speed-Vacuum, Thermo) tryptic digests re-suspended in 2% (v/v) acetonitrile (ACN) 1% (v/v) formic acid were desalted and concentrated by C18 microcolumns (OMIX tips, Agilent Technologies, Santa Clara, CA, USA) following the manufacturer’s instructions.

### Liquid chromatography tandem mass spectrometry assays

Dried peptide mixtures were re-suspended in 2% ACN in 0.1% FA, and centrifuged for 5 min at 14,000 × *g* to remove insoluble material. Samples were resolved by liquid chromatography (nanoLC Ultra 2D, Eksigent^®^) on a MicroLC column ChromXP™ (300 µm ID × 15 cm length, 3 µm particles, 120 Å pore size, Eksigent^®^) at 5 µL/min with a multistep gradient: 0–2 min linear gradient from 2 to 5%, 2–45 min linear gradient from 5 to 30% and, 45–46 min to 35% of ACN in 0.1% formic acid and 5% dimethyl sulfoxide, and analyzed on a TripleTOF^®^ 5600 System (ABSciex^®^). Data acquisition was performed using information-dependent acquisition (IDA) and Sequential Window Acquisition of All Theoretical Mass Spectra (SWATH-MS) of each individual replicate (Anjo et al. 2015). For IDA experiments, the mass spectrometer was set to scan full spectra (*m/z* 350–1250), followed by up to 100 MS/MS scans (*m/z* 100–1500). Candidate ions with a charge state between +2 and +5 and counts above a minimum threshold of 10 counts/s were isolated for fragmentation and one MS/MS spectra was collected before adding those ions to the exclusion list (mass spectrometer operated by Analyst^®^ TF 1.7, ABSciex^®^). Rolling collision was used with a collision energy spread (CES) of 5. For SWATH-MS-based experiments, the mass spectrometer was operated in a looped product ion mode (Gillet et al. 2012) and a set of 60 windows (see Online Resource 1) of variable width (containing a *m/z* of 1 for the window overlap) was constructed covering the precursor mass range of *m/z* 350–1250. A 250-ms survey scan (*m/z* 350–1500) was acquired at the beginning of each cycle and SWATH MS/MS spectra were collected from m/z 100–1500 for 50 ms resulting in a cycle time of 3.25 s. The collision energy applied to each *m/z* window was determined considering the appropriate collision energy for a +2 ion centered upon this window and the CES was also adapted to each m/z window.

### Proteome data processing

Protein identification from the IDA experiments was performed using ProteinPilot™ software (v5.1, ABSciex^®^) with the following parameters: (i) search against a database composed by all the entries for the echinoderm phylum from NCBI (https://www.ncbi.nlm.nih.gov/, release at July 2016, 60358 protein entries), *Patiria miniata* annotated genome (http://www.echinobase.org/Echinobase/, 29805 protein entries), and the sequence of the internal standard (MBP-GFP); (ii) iodoacetamide alkylated cysteines as fixed modification; (iii) trypsin as digestion type. An independent false discovery rate (FDR) analysis using the target-decoy approach provided with ProteinPilot™software was used to assess the quality of the identifications, and confident identifications were considered when identified proteins and peptides reached a 5% local FDR (Sennels et al. 2009). The results from the three biological replicates were manually aligned to consistently select the same isoform in all replicates. The criteria used were (i) selection of the isoforms presented in the higher number of replicates, and (ii) arbitrary selection of one isoform from the groups of isoforms identified in the same number of replicates. Additionally, a specific library of precursor masses and fragment ions was created by performing a single search as described above using all the files from the IDA experiments. This library was used to perform protein identification via SWATH-MS data (Teixeira et al. 2017). Briefly, the chromatographic profiles of the peptides presented in the library were extracted from the SWATH-MS data using the SWATH™ processing plug-in for PeakView™ (v2.0.01, AB SCIEX) with an extracted-ion chromatogram (XIC) window of 4 min. Peaks were extracted for up to 5 target fragment ions of up to 15 peptides per protein. Confident identification was considered for proteins with at least one peptide with an FDR below 1%. Proteins identified following the defined acceptance criteria in at least one of the three biological replicates with one of the methods (IDA or SWATH) were considered for posterior analyses.

The mass spectrometry and proteomics dataset are available through the ProteomeXchange Consortium via the PRIDE partner repository, under dataset identifiers PXD027009 and 1ssss0.6019/PXD. (The reviewers may access the currently private dataset using reviewer_pxd027009@ebi.ac.uk as Username and Hw17Effb password.)

### Bioinformatics analyses

The limited number of echinoderm proteins deposited on the available protein sequence databases can impair the success of data interpretation, so the identified proteins were further submitted to protein–protein Basic Local Alignment Search Tool (BLASTp, http://www.uniprot.org/blast/), against UniProt knowledgebase (UniProtKB) database search available on the UniProt website (http://www.uniprot.org), in order to carry out a homology-driven proteome characterization of *M. glacialis* CE without taxonomic restrictions. The Software Tool for Rapid Annotation of Proteins (STRAP) was used to fully annotate the identified proteins using the UniProt and EBI QuickGO (http://www.ebi.ac.uk/QuickGO/) gene ontology information (Bhatia et al. 2009). A Search Tool for the Retrieval of Interacting Genes/Proteins (STRING) (Szklarczyk et al. 2015), with a confidence score above 0.900, functional network construction was performed after a BLASTp search of the identified proteins against *Strongylocentrotus purpuratus* entries from UniProtKB database search. Moreover, to have additional information about the pathways to which the proteins of the predicted networks belong, Kyoto Encyclopedia of Genes and Genomes (KEGG) pathway tool was used (http://www.genome.jp/kegg/pathway.html).

## Results

### Ultrastructural analysis of coelomic epithelium in non-regenerating arms

**Fig. 1:**
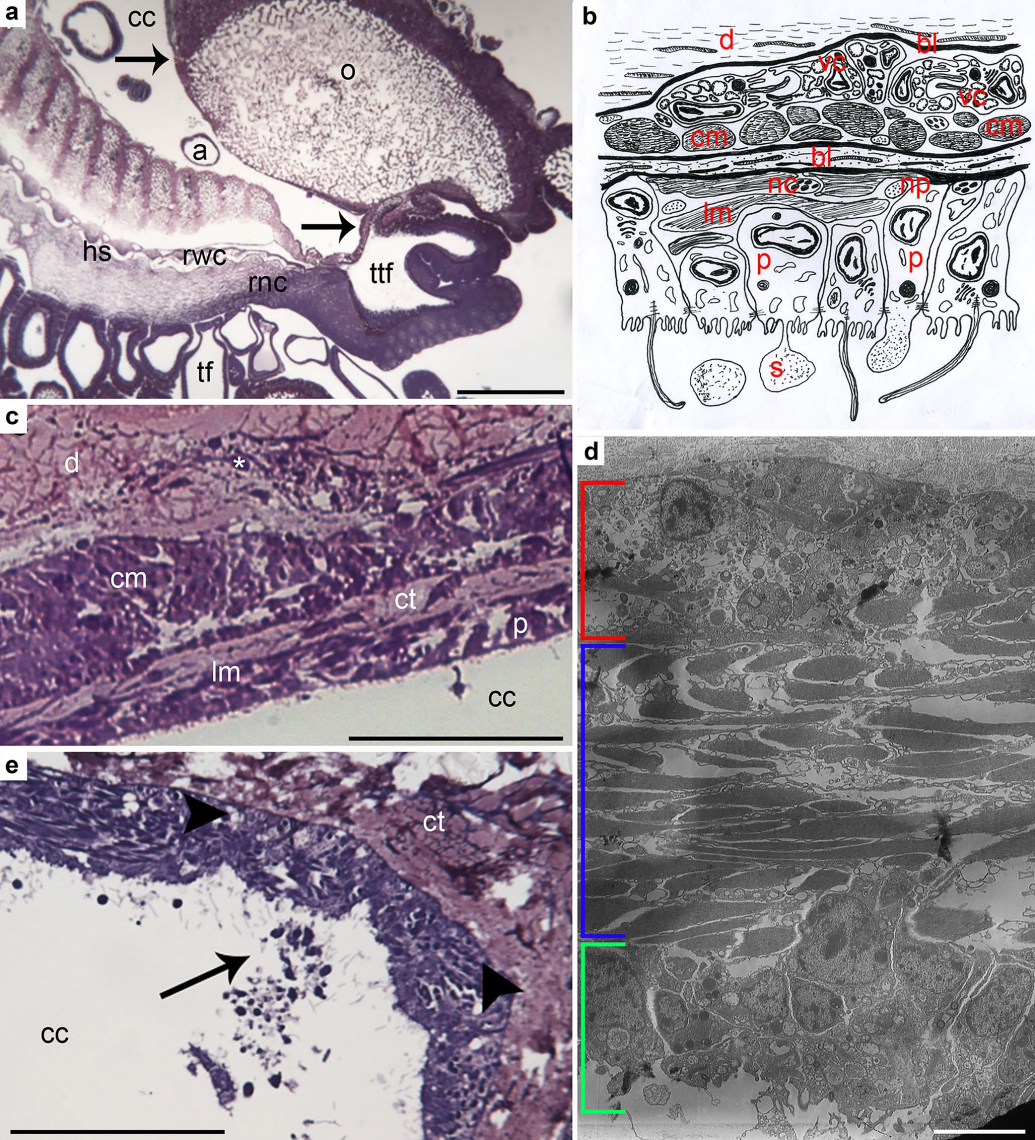
**a**–**e** CE of non-regenerating arm. **a** Semithin longitudinal section of *M. glacialis* non-regenerating arm tip showing coelomic compartments lined by CE (arrows): perivisceral coelomic cavity (cc), radial water canal (rwc), ampullae (a), tube feet (tf), hyponeural sinus (hs), terminal tube foot (ttf). Scale bar = 400 µm. **b** Diagrammatic representation of perivisceral CE structure consisting of apical peritoneocytes (p); longitudinally oriented subapical myocytes (lm); common basal lamina (bl); basiepithelial nerve processes (np); and neurosecretory cells (nc). Circular muscle layer (cm) is housed within the dermis (d) and is surrounded by a proper basal lamina (bl). Unusual vesicular cells (vc) are detected close to circular myocytes: they project their flagella into intercellular niches. Note the apocrine secretion(s) of peritoneocyte apical portions. **c** Semithin longitudinal section of proximal CE showing peritoneocyte layer (p); longitudinal (lm); and circular (cm) muscle layers separated by connective tissue (ct); dermis (d) infiltrated by muscle bundles (asterisk). Scale bar = 10 µm. **d** TEM micrograph of the distal CE showing peritoneocytes (green); longitudinal muscle layer (blue); vesicular cell layer (red). Note the absence of the circular muscle layer. Scale bar = 2 µm. **e** Semithin longitudinal section of the distal CE showing peritoneocyte layer releasing blebs (arrow); longitudinal muscle layer; vesicular cell layer (arrowheads). Scale bar = 10 µm. Abbreviations: CE, coelomic epithelium; ct, connective tissue; o, ossicles; rnc, radial nerve cord; TEM, transmission electron microscopy

The perivisceral CE of *M. glacialis* arm (Fig. 1a) displayed the typical bi-layered structure of a pseudostratified myoepithelium described in the literature for other starfish (Wood and Cavey 1981; Rieger and Lombardi 1987), where apical squamous/cubic peritoneocytes and subapical longitudinally oriented myocytes are placed on the same basal lamina (Fig. 1b–d). A thin layer of connective tissue separated the myoepithelial components from an inner, deeper layer of circular (i.e., transversally oriented) myocytes (Fig. 1b–d), this latter being apparently lacking in the distal-most arm tip (Fig. 1e).

**Fig. 2:**
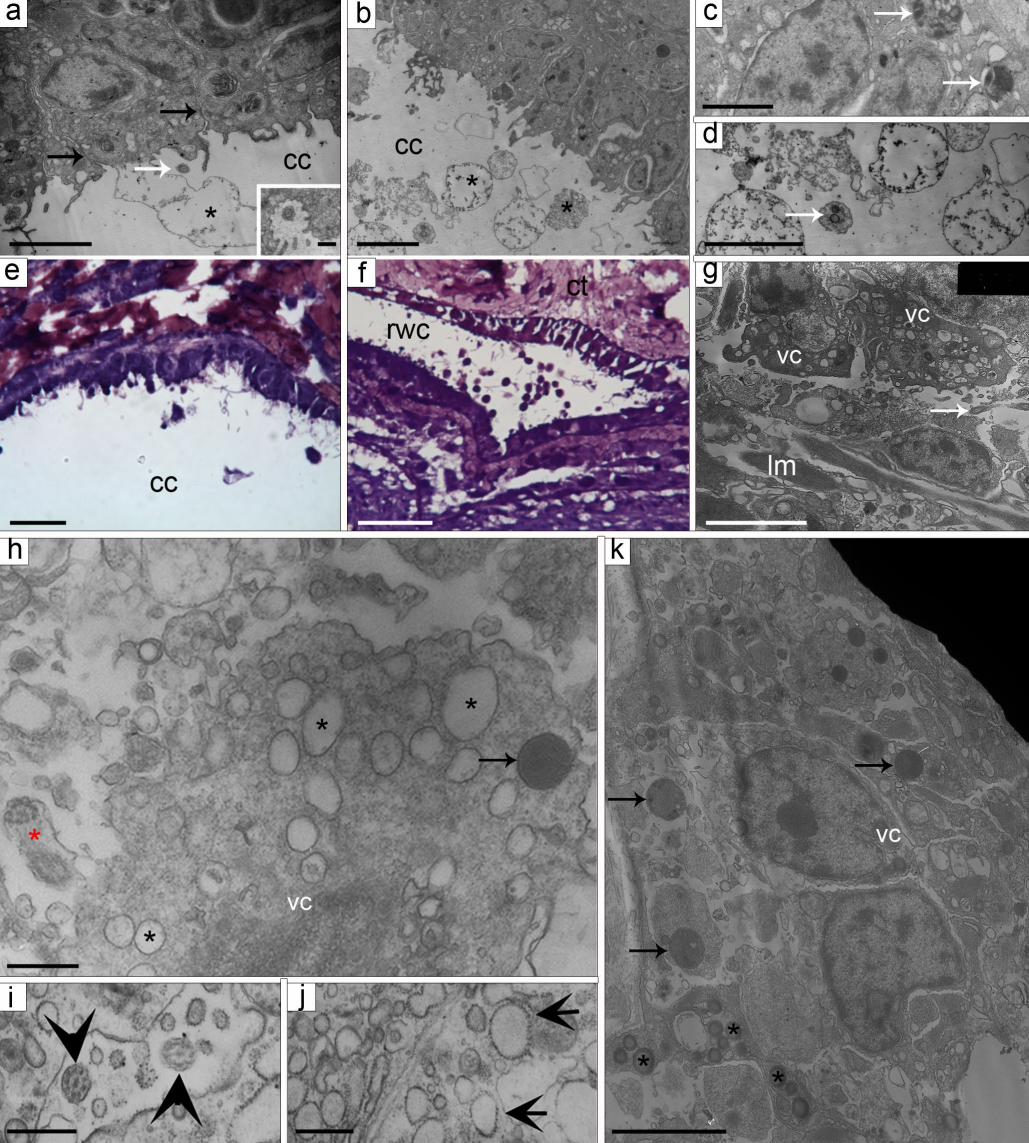
**a**–**k** CE of non-regenerating arm. **a** TEM micrograph of peritoneocytes. Apical junctions among adjacent cells are visible (black arrows) as well as typical flagella (white arrow). Intense apocrine secretion (asterisk) can be observed. Inset: detail of flagellum surrounded by a crown of microvilli. Scale bar A = 2 µm, scale bar inset = 500 nm. **b** TEM micrograph of peritoneocytes. Secreted cytoplasmic portion of different shapes and sizes (asterisks) are visible in the perivisceral coelomic lumen. Scale bar = 2 µm. **c** TEM micrograph of peritoneocytes. Phagosomes are visible at the cell base (arrows). Scale bar = 1 µm. **d** TEM micrograph of secreted peritoneocyte products filled with granular electron-dense material and other small vesicles (arrow). Scale bar = 2 µm. **e** Semithin longitudinal section of perivisceral CE showing an evident apocrine secretion. Scale bar = 5 µm. **f** Semithin longitudinal section of radial water canal CE: a massive apical secretion is shown. Scale bar = 10 µm. **g** TEM micrograph of CE vesicular cell layer. Vesicular cells are characterized by electron-lucent vesicles and flagella (arrow), and are in direct contact with the longitudinal muscle layer (lm). Scale bar = 2 µm. **h** TEM micrograph of vesicular cell containing small RER cisternae (black asterisks) and a presumptive phagosome (arrow). Note the presence of flagellum (red asterisk). Scale bar = 1 µm. **i** TEM detail of flagella of vesicular cells (arrowheads) projecting in an intercellular niche. Scale bar = 1 µm. **j** TEM detail of vesicular cell showing RER cisternae (arrows). Scale bar = 1 µm. **k** TEM micrograph of vesicular cells containing phagosomes (arrows) and presumptive lipid droplets (asterisks). Scale bar = 2 µm. Abbreviations: cc, coelomic cavity; CE, coelomic epithelium; RER, rough endoplasmic reticulum; rwc, radial water canal; TEM, transmission electron microscopy; vc, “vesicular” cells

TEM analysis confirmed that the peritoneocytes are highly differentiated polarized cells with an apical flagellum surrounded by a crown of microvilli (Fig. 2a). Their cytoplasm was characterized by several small electron-lucent or finely granular roundish vesicles, different in shapes and sizes, occasional phagosomes, myelin figures, and presumptive lipid droplets (Fig. 2a–d), typical junctional complexes between adjacent peritoneocytes being recognizable apically (Fig. 2a). Peritoneocytes were massively involved in peculiar phenomena of apocrine secretion (Fig. 2a, b, d), through which the apical portions of these cells (including cytoplasm and cell membrane) were released into the coelomic lumen. The released cytoplasmic portions (0.6–3 µm in diameter) had slightly irregular roundish shapes, and contained granular and moderately electron-dense material and occasionally also electron-lucent vesicles or inclusions (Fig. 2a, b, d). Once released by the cells, they were soon disaggregated and not detectable any more in the coelomic lumen. This phenomenon was visibly concentrated in the distal-most tip of the arm. Although also present in the perivisceral coelom (Fig. 2e), it was most evident in the radial water canal CE, particularly at the level of the terminal podium (Fig. 2f), whereas in the rest of the arm it was rarely detectable.

As shown by ultrastructural observations, a thick layer of rather uncommon “vesicular” cells was present at the borders of the inner layer of transversally oriented myocytes. These “vesicular” cells and myocytes did not form distinct layers, as no proper basal lamina separated them (Fig. 2g). “Vesicular” cells showed euchromatic nuclei and were characterized by a massive cytoplasmic presence of swollen rough endoplasmic reticulum (RER) cisternae (Fig. 2h), and few phagosomes (Fig. 2h, k). Interestingly, adjacent vesicular cells often formed together intercellular niches bordered by microvillar projections and hosting flagella (Fig. 2g, h). Due to the cytological complexity of these cells or cellular aggregates, it was not always possible to establish the specific cellular element originating the flagellum nor the presence of single or multiple flagella on the same cell. Scattered among these vesicular cytotypes, other cells containing phagosomes and presumptive lipid droplets (Fig. 2k) were occasionally detected as well, together with neuronal or presumptive neurosecretory processes. In the distal-most arm tip where the circular muscle layer was not present, the vesicular layer was directly in contact with the longitudinal muscle layer (Fig. 2g).

There was no direct evidence of active cell release from the CE nor of any presence of morphologically undifferentiated cells throughout the CE.

### Ultrastructural analysis of coelomic epithelium in regenerating arms

**Fig. 3:**
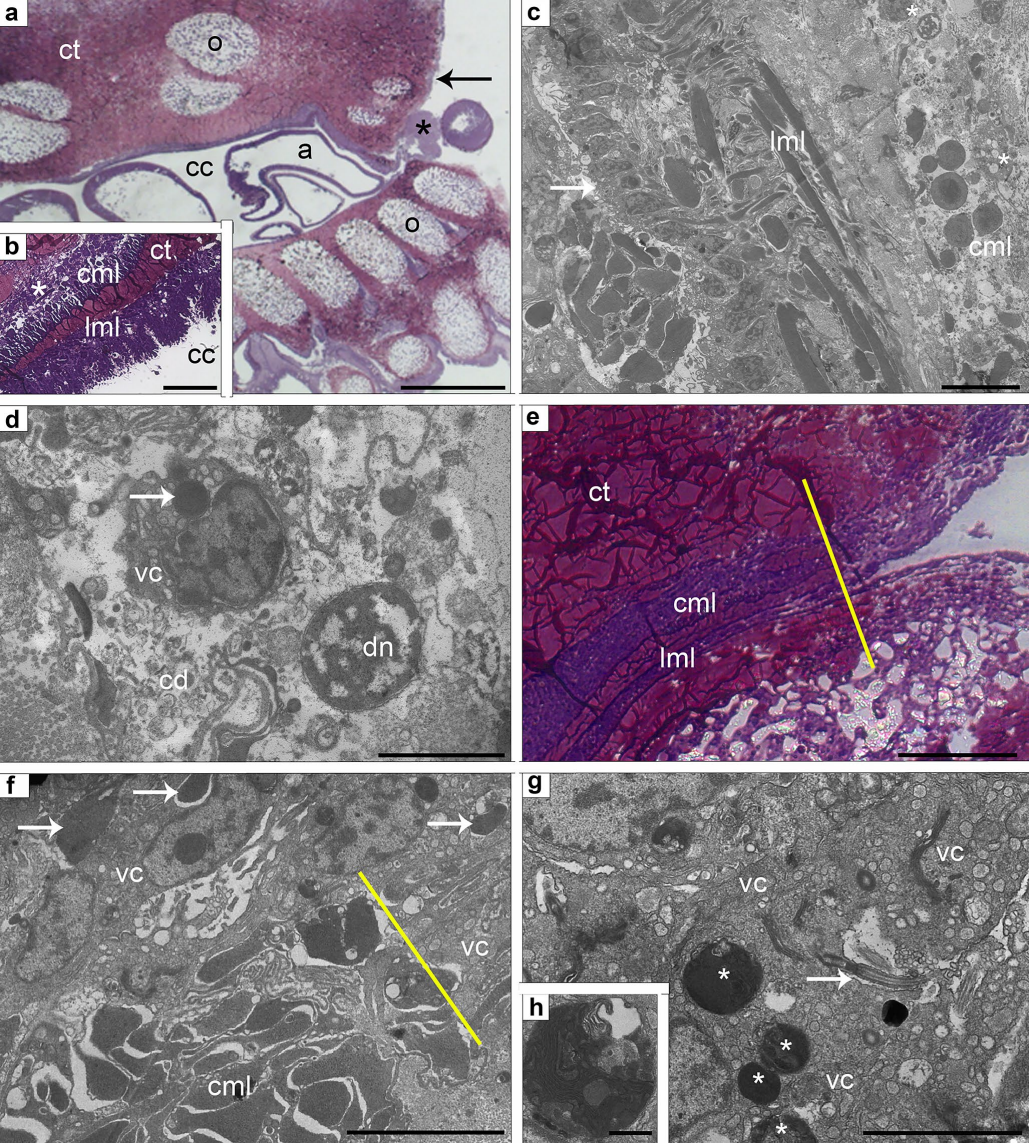
**a**–**h** CE of stump in a regenerating sample: 24–48 h p.a. **a** Semithin longitudinal section of *M. glacialis* arm tip at 24 h p.a. Note the presence of coelomocyte clots at the level of the wound (asterisk), the thickening of the perivisceral CE immediately close to the amputation site, and the new thin epithelium covering the wound edges (arrow). Scale bar = 400 µm. **b** Semithin longitudinal section of the regenerating CE at 24 h p.a. showing peritoneocyte layer with massive apical secretion; longitudinal muscle layer (lml); thickening of vesicular cell layer (asterisk) associated to circular muscle layer (cml); connective tissue layer (ct). Scale bar = 10 µm*.* **c** TEM micrograph of regenerating CE at 24 h p.a. showing peritoneocytes (arrow); longitudinal muscle layer (lml); circular muscle layer (cml) with first signs of rearrangement; vesicular cells (asterisks). Scale bar = 2 µm. **d** TEM micrograph showing details of vesicular cell layer. A degenerating nucleus (an) is visible among cell debris (cd); a vesicular cell contains a phagosome (arrow). Scale bar = 2 µm. **e** Semithin longitudinal section of the regenerating CE at the level of the wound closure. The yellow line indicates the beginning of the looser CE structure with the progressive disappearing of both longitudinal (lml) and circular (cml) muscle layers. Scale bar = 50 µm*.* **f** TEM micrograph of circular muscle layer (cml). Fibers undergo rearrangement phenomena indicated by evident modifications of their contractile apparatus (arrows). Flagellated vesicular cells are apparently involved. Yellow line marks the rearrangement beginning in both longitudinal and circular muscle fibers. Scale bar = 5 µm*.* **g** TEM micrograph of vesicular cells showing flagella (arrow) projecting into intercellular niches. Big phagosomes are visible (asterisks). Scale bar = 2 µm*.* (h) TEM detail of a large phagosome in vesicular cell. Scale bar = 1 µm. Abbreviations: a, ampulla; cc, coelomic cavity; CE, coelomic epithelium; o, ossicle; p.a., post amputation; TEM, transmission electron microscopy; vc, “vesicular” cell

At 24 h p.a., the perivisceral CE close to the wound area was apparently thicker than that in non-regenerating conditions (Fig. 3a, b), with peritoneocytes presenting in particular a higher concentration of electron-lucent vesicles and phagosomes in their apical cytoplasmic portion. An overall increase of apocrine secretion phenomena appeared to involve peritoneocytes along the whole CE of the stump (Fig. 3a, b). The muscle layers (particularly the circular one) were disorganized and underwent remodeling: myofibers displayed a highly irregular shape, their contractile apparatus showed signs of condensation, and numerous cell debris were widespread between disarranged myocytes as well as few apoptotic nuclei (Fig. 3c, d, f). Several “vesicular cells” containing a consistent number of inclusions, mainly phagosomes, were visible (Fig. 3c, d, f–h).

At 48 h p.a., the perivisceral CE distal to the amputation plane showed the same overall ultrastructure described at 24 p.a., the only relevant difference being the reduced thickness of the “vesicular” cell layer. Apocrine secretion phenomena were still present, particularly in the distal-most part of the stump CE, although apparently less massive than in the previous stage (see Online Resource 2). At the level of the wound closure, perivisceral CE peritoneocytes displayed heterochromatic and irregular nuclei, high number of electron-lucent vesicles, and large phagosomes (Fig. 3g, h). The underlying muscle layers underwent an intense reorganization and remodeling. As a result, first the circular layer and later the longitudinal layer showed a proximo-distal reduction and, at the distal-most arm tip, completely disappeared (Fig. 3e). Here, CE peritoneocytes were directly in contact with the vesicular layer, whose cells contained numerous and big phagosomes, small roundish inclusions, and myelin figures (Fig. 3g).

**Fig. 4:**
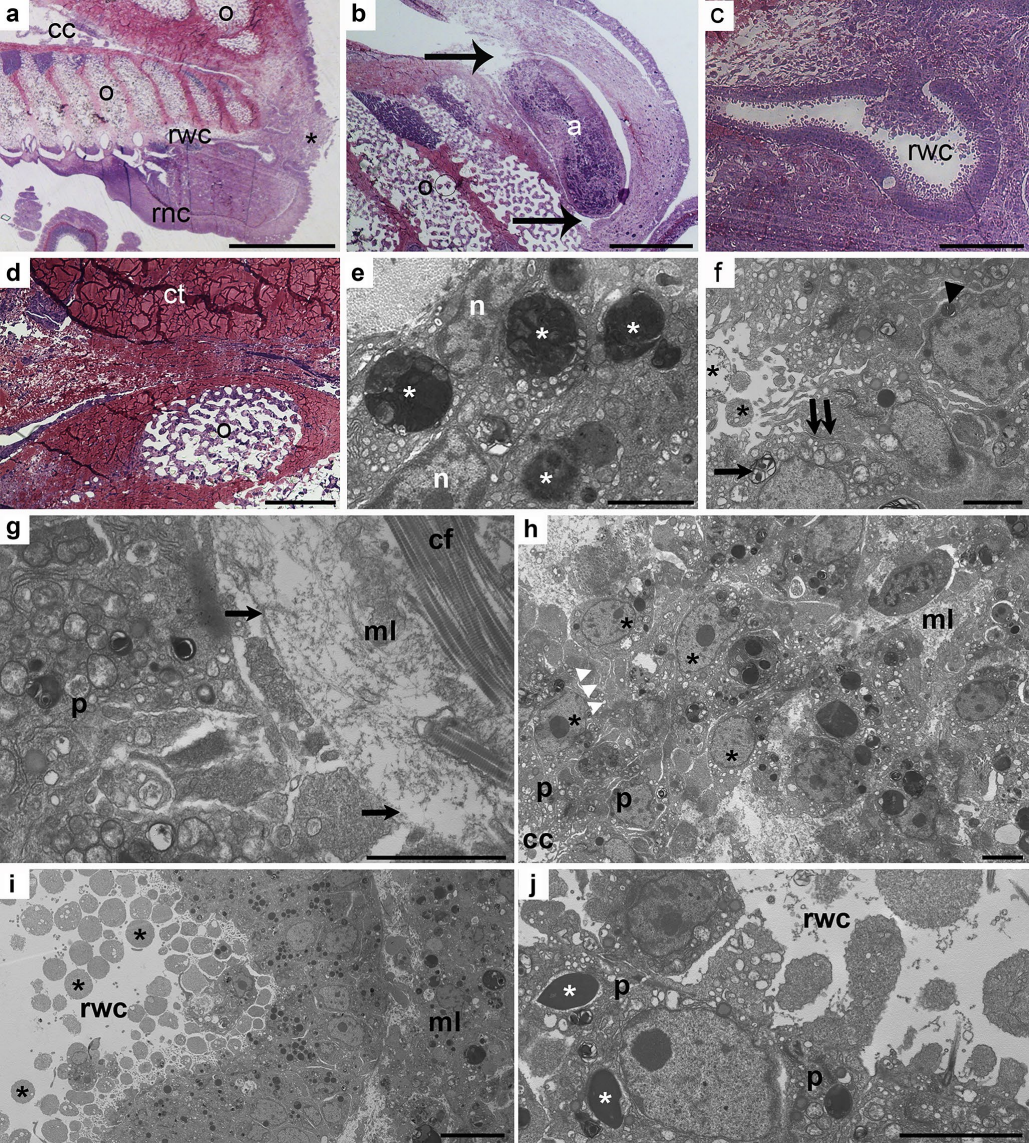
**a**–**j** CE of 1 week p.a. regenerating sample. **a** Semithin longitudinal section of *M. glacialis* regenerating arm tip. Re-growing perivisceral coelomic cavity (cc) and radial water canal (rwc) appear to penetrate an area of loose coelomocytes clot (asterisk) at the level of the wound site. Scale bar = 400 µm. **b** Semithin longitudinal section of the regenerating perivisceral coelomic cavity (arrows). A rearranging ampulla (a) is recognizable. Scale bar = 50 µm. **c** Semithin longitudinal section of the regenerating radial water canal (rwc) lined by the CE. Note the peritoneocyte apocrine secretion at the very tip of the new regenerating structure. Scale bar = 20 µm. **d** Semithin longitudinal section of CE at the level of the closure of the wound. Both longitudinal and circular muscle layers are not detectable. Scale bar = 50 µm. **e** TEM micrograph of vesicular cell layer showing several big phagosomes (asterisks). Scale bar = 2 µm. **f** TEM micrograph of the new coelomic cavity tip, showing details of peritoneocytes. Apical secretion phenomena are evident (asterisks). Adjacent cells are apically joined by cell junctions (double arrows); numerous myelin figures (arrow) and phagosomes (arrowhead) are present in their cytoplasm. Scale bar = 2 µm. **g** TEM micrograph of regenerating perivisceral CE (p). A faint basal lamina (arrows) is present under coelothelial cells (p) and separates them from the underlying mesenchymal tissue. Scale bar = 2 µm. **h** TEM micrograph of regenerating perivisceral CE (p) showing evident cell migration toward the mesenchymal tissue (mt). Triple white arrowheads indicate the site of EMT. Cells have high nucleus/cytoplasm ratio (asterisks). Scale bar = 2 µm. **i** TEM micrograph of new radial water canal (rwc) CE. Peritoneocytes are releasing apical cytoplasmic portions (asterisks). Scale bar = 5 µm. **j** TEM micrograph of radial water canal (rwc) CE showing the presence of SLSs (asterisks) in peritoneocytes (p). Scale bar = 2 µm. Abbreviations: CE, coelomic epithelium; cf, collagen fibrils; ct, connective tissue; ml, mesenchymal layer; n, nucleus; o, ossicle; p.a., post amputation; rnc, radial nerve cord; SLS, spindle-like structures; TEM transmission electron microscopy

At 1 week p.a., both the perivisceral coelomic cavity and radial water canal (RWC) formed highly hypertrophic outgrowths penetrating the regenerating bud (Fig. 4a–c). In the distal part, both the longitudinal and the circular muscle layers were still lacking (Fig. 4d). The “vesicular cells” displayed larger phagosomes if compared to the previous stage, often including rests of disorganized contractile apparatus of myocytes (Fig. 4e). The newly formed perivisceral CE was composed only by a monolayer of cubic peritoneocytes (Fig. 4f) basally hosting a highly euchromatic nucleus and apically joined to each other by typical junctional complexes. These cells appeared scarcely differentiated, although bearing an apical flagellum and containing numerous electron-lucent vesicles, occasional large phagosomes, and myelin figures: in addition, at the distal-most tip of the new coelomic cavity, they clearly showed intense phenomena of apical secretion (Fig. 4f). At this level, the peritoneocytes were supported only by a discontinuous and very faint basal lamina (Fig. 4g). Here, interestingly, signs of epithelial-mesenchymal transition could be observed (Fig. 4h): coelothelial cells apparently detached from the basal part of the epithelium and migrate in the underlying mesenchymal tissue. These cells presented large euchromatic nuclei and high nucleus/cytoplasm ratios. In contrast to perivisceral coelom, radial water canal CE presented a well-defined basal lamina (Fig. 4j). Epithelial cells displayed a high number of phagosomes, in some cases including condensed contractile apparatus organized in typical spindle-like structures (Fig. 4j). As for the perivisceral CE, peritoneocytes of the arm tip were apparently involved in massive apical secretion (Fig. 4i).

No direct release of free wandering coelomocytes from CE was observed in any of the considered time-points.

### Protein identification, BLASTp searches, and gene ontology annotation of non-regenerating CE

**Fig. 5:**
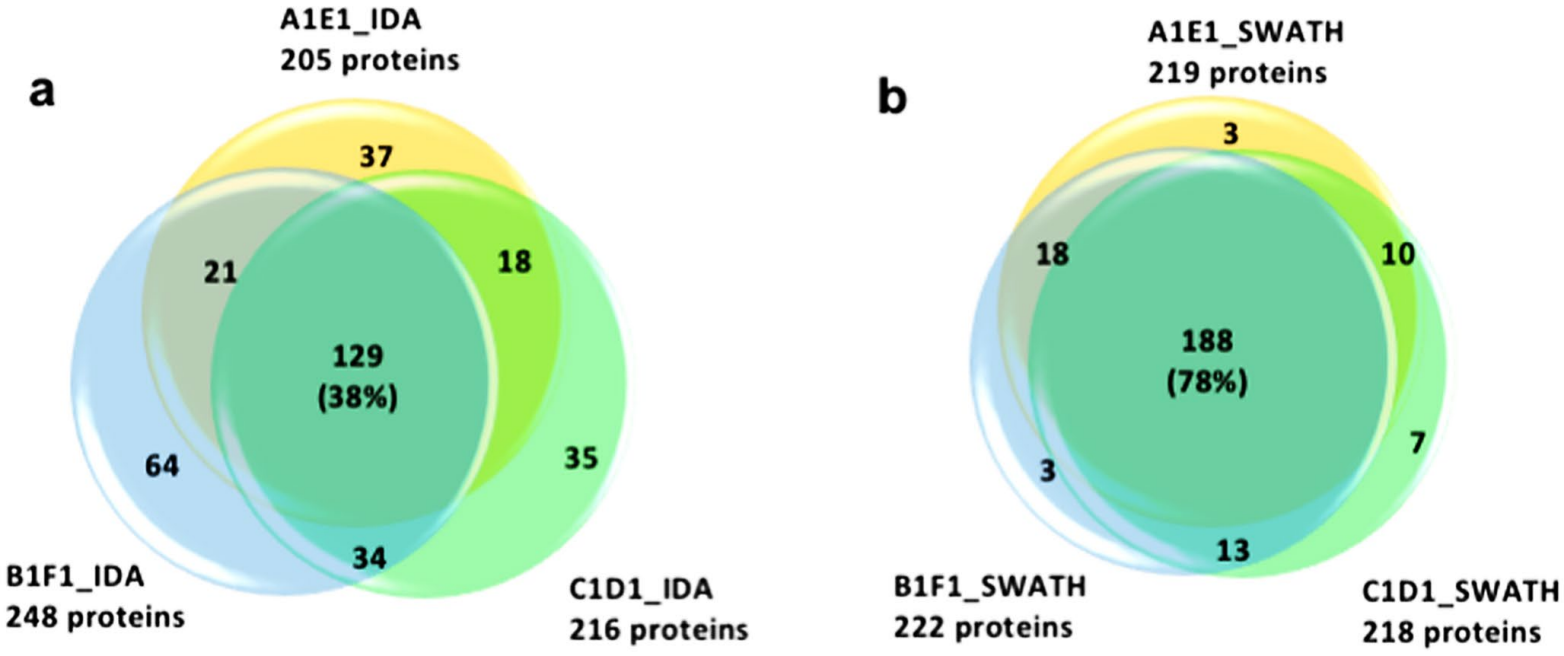
Proteins identified in the *M. glacialis* CE by LC–MS/MS (**a**, **b**). **a** IDA analysis results; **b** SWATH analysis results. Each colored circle corresponds to one of the three biological replicates analyzed by LC–MS/MS: in pink, number of proteins identified from A1E1 replicate; in green, number of proteins identified from B1F1 replicate; in blue, number of proteins identified from C1D1 replicate. The total number of proteins identified for each biological replicate is shown between parentheses

From the analysis of the three biological replicates (A1E1, B1F1, C1D1), 345 proteins were identified in at least 2 biological replicates. In particular, from IDA analysis, 338 proteins were identified (Fig. 5a, Online Resource 3), whereas from SWATH analysis (Fig. 5b, Online Resource 3), 242 proteins were identified, with 173 proteins in common for the two liquid chromatography tandem mass spectrometry (LC–MS/MS) acquisition methods. From the total of 345 proteins identified, 227 were identified from *P. miniata*, 80 from *S. purpuratus*, and 38 from other echinoderms. From BLASTp, we obtained a consistent number of identified proteins homologous to echinoderm proteins deposited on the searched protein databases (32%). However, being a homology-driven proteomics characterization, several of the identified proteins from the starfish CE were homologous to proteins from other organisms (i.e., Chordata 28.5%; Arthropoda 18.5%; Mollusca 11.5%; other marine invertebrates 9%; complete list of characterized proteins in Online Resource 4). The full annotation of the identified proteins using the UniProt Gene Ontology information by STRAP analysis is provided in Online Resource 5.

**Fig. 6:**
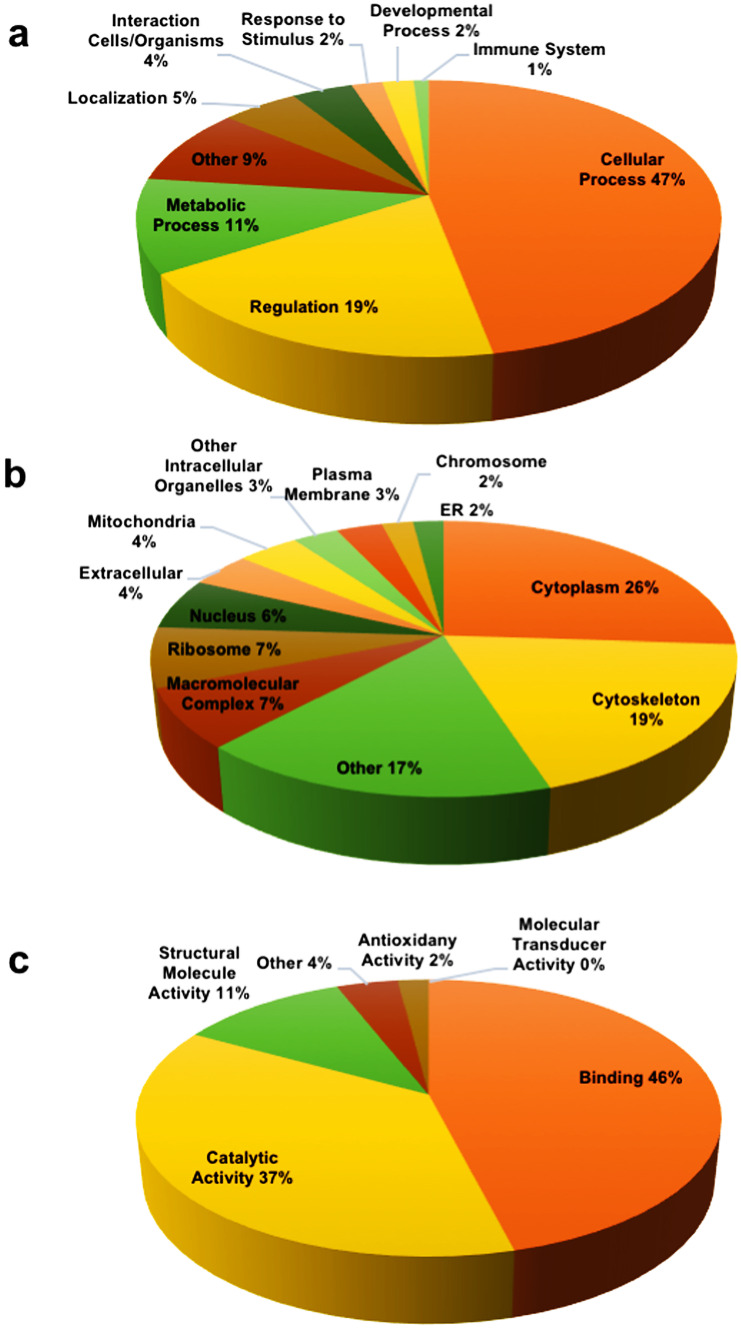
Gene ontology results from STRAP analysis (**a**–**c**). **a** Biological process; **b** cellular components; **c** molecular function

The biological processes attributed to the majority of the identified proteins in the CE (Fig. 6a) were cellular (47%), were metabolic (11%), and in regulation (19%). The remaining proteins were involved in localization (5%), interaction with cells and organisms (4%), response to stimulus (2%), developmental process (2%), and immune system (< 1%), whereas 9% of the proteins had no identified biological processes. Most identified proteins are cytoplasmic (26%) or cytoskeletal (19%) (Fig. 6b). Considering the molecular function (Fig. 6c), proteins having a binding function and a catalytic activity were highly represented (46% and 37%, respectively).

A STRING analysis was performed for the identified proteins homologous to *S. purpuratus* genome (285 proteins), aiming for a deeper understanding of the physiological and biological functions of the CE. Applying the higher confidence restriction (> 0.7) and excluding the general metabolism clusters, 11 networks of predicted functional associations, comprising 53 proteins, were defined (Online Resource 6). Through analysis of KEGG pathway ontology for these networks, we found an enrichment in proteins relevant for maintaining epithelial tissue structure and functions (namely cell–cell adhesion and membrane integrity), and involved in membrane trafficking processes (like exo- and endocytosis), cell and ciliary motility, protein processing, exporting, and degradation functions (Table 1). From these proteins, 27 were related to exosomal processes. Exosomes are described as small endocytic vesicles released into the extracellular environment that mediate a variety of physiological and pathological processes. Interestingly, proteins associated to phagocytosis, the main biological function traditionally attributed to coelomocytes, were also well represented.

**Table 1:** Non-carbon metabolism KEGG pathways of *M. glacialis* CE identified proteins, their detection in the CE of other asteroidea and in *M. glacialis* coelomocytes by biological process

**Protein code**	**Protein ID**	**CE** (Gabre et al. 2015)	**CE** (Sharlaimova et al. 2021)	**Coelomocytes** (Franco et al. 2011)
Membrane trafficking				
SPU_000035	EF-hand domain-containing protein D2			X
SPU_012228	Actin related protein 2/3 complex, subunit 4		X	X
SPU_018717	Actin-related protein 2*		X	X
SPU_017502	Actin related protein 2/3 complex, subunit 2		X	X
SPU_010631	Actin-related protein 3*	X		X
SPU_021007	Capping protein (actin filament) muscle Z-line, beta*	X		
SPU_027974	Actin related protein 2/3 complex, subunit 1A/1B*			X
SPU_010545	Ras-related C3 botulinum toxin substrate 1*			
SPU_002616	Clathrin light chain A*	X		X
SPU_026199	Clathrin light chain A*			X
SPU_011029	GTPase_Ras_Rho			
SPU_005032	Calmodulin*			
SPU_021511	Calmodulin*			
Cell–cell adhesion				
SPU_020920	Actinin alpha 1/4*	X		X
SPU_001935	Vinculin*			X
SPU_022771	Lipoma-preferred partner			
SPU_026137	Catenin alpha			
SPU_000241	Talin*			
Phagocytosis				
SPU_006211	Peroxiredoxin 2/4*			X
SPU_006214	Superoxide dismutase (Cu–Zn)			
SPU_014869	Peroxiredoxin 1*	X		
SPU_013368	Peroxiredoxin 6	X		X
SPU_019087	Ras-related protein Rab-7A*		X	
SPU_005779	Ras-related protein Rap-1A			
SPU_004785	Rab GDP dissociation inhibitor*	X		
SPU_012112	Ras-related protein Rab-5B		X	
SPU_010762	Ras-related protein Rab-1A*			
XP_030839096	Scavenger receptor cysteine-rich domain superfamily			
SPU_010545	Rho-related protein racB			
SPU_019494	Cell division control protein 42*	X	X	
SPU_019715	Mitogen-activated protein kinase			
Plasma membrane integrity				
SPU_026521	Adducin			
SPU_011699	Spectrin alpha			
Cell motility				
SPU_010054	Myosin-16 isoform X5			
SPU_021293	IQ motif containing GTPase activating protein 1*		X	
SPU_013183	Troponin I-like isoform X1	X	X	
SPU_001613	Myosin*	X		
SPU_007146	Ras GTPase-activating-like protein IQGAP1*			
SPU_025244	Myosin*	X	X	
Ciliary motility				
SPU_005442	Radial spoke head protein 9			
SPU_006699	Dynein intermediate chain 2, axonemal	X		
Protein processing in endoplasmic reticulum and export				
SPU_010434	Calreticulin	X		
SPU_008560	Endoplasmic reticulum chaperone BiP*	X		X
SPU_024103	Chaperonin GroEL*	X	X	X
SPU_018964	FK506-binding protein 2		X	
SPU_028874	Cyclophilin B			
SPU_004131	Vesicle-associated membrane protein A			
SPU_027527	Heat shock protein gp96			
SPU_024221	Sorting nexin-1/2			X
Ubiquitin proteosome system				
SPU_015276	Ubiquitin-large subunit ribosomal protein L40e			X
SPU_024507	S-phase kinase-associated protein 1	X		
SPU_018598	Ubiquitin-conjugating enzyme E2 N	X		
SPU_012828	Ubiquitin-conjugating enzyme E2 L3			
SPU_009521	Ubiquitin-conjugating enzyme E2 D		X	
SPU_016382	E3 ubiquitin-protein ligase TRIM23			
SPU_023286	20S proteasome subunit alpha 6*		X	
SPU_023663	Histone H2A*			X

^*^Proteins related to exosomal processes

## Discussion

In this work, we characterized the submicroscopic anatomy and proteome of both non-regenerating (homeostatic) and regenerating arms of the starfish *Marthasterias glacialis*, with the aim of unraveling the following issues: (1) is starfish CE an active source of free wondering coelomocytes? (2) Is the CE a source of multipotent (stem) cells during regeneration? (3) Does the CE display other key functions involved in regeneration as well as in overall animal physiology?

To address these questions, we carried out an integrated analysis of this complex tissue by combining detailed microscopic and ultrastructural analyses with modern proteomics methods. We compared the histological results obtained in standard physiological condition, i.e., in intact normal arms, with those obtained in post-traumatic regrowth condition, i.e., in regenerating arms at different stages. Although it was desirable to add to these results a differential comparison of regenerating CE proteome, two aspects have prevented it: (1) the difficulty of establishing a procedure, using a compatible method, to monitor the portion of CE to be harvested; (2) the huge number of animals needed to extract the CE at the boundary of the regenerating arm tip.

### Coelomic epithelium as source of circulating coelomocytes

Several authors consider echinoderm CE as the main source of free wandering coelomocytes, a function often referred as “hematopoiesis” (Muñoz-Chápuli et al. 2005; Holm et al. 2008; Sharlaimova et al. 2021). These circulating elements are considered the immune cells involved in the clearance of foreign materials, pathogens, and cell debris, as well in haemostatic functions, since they form clots at the wound sites (Pinsino et al. 2007; Smith et al. 2010; Sharlaimova et al. 2014; Ben Khadra et al. 2015). *M. glacialis* coelomocytes have been recently characterized by means of different cytometry/microscopy (Andrade et al. 2021) and proteomic approaches (Franco et al. 2011), which partially shed light on this heterogeneous cell population and its possible related functions in this species. According to Sharlaimova et al. (2021), in the starfish *Asterias rubens*, the CE contains undifferentiated precursors which contribute to the replenishment of at least some sub-populations of circulating coelomocytes, the so-called agranulocytes. In the present work, we never observed a direct release of free wandering coelomocytes from the apical part of the CE toward the coelomic lumen, either in homeostatic non-regenerating or in regenerating conditions. Nevertheless, we observed shared ultrastructural features between the specific *M. glacialis* thrombocyte-like coelomocyte sub-population (Andrade et al. 2021) and peritoneocytes. Indeed, they both possess particular cytoplasmic features, including electron-lucent vesicles and vacuoles as well as electron-dense granules and phagosomes (see below for further considerations). An ultrastructural similarity between CE cells and circulating coelomocytes has been reported several times (Holm et al. 2008; Gorshkov et al. 2009; Sharlaimova et al. 2010) and our observations confirm this morphological analogy, thus providing further support to the hypothesis of a coelothelial origin of this cell sub-population.

Overall, proteins specific to “hematopoiesis” were not found, this result being explainable by the presence in the total protein extract analyzed by LC–MS/MS of highly abundant proteins, which hampers the identification of lower abundant proteins. Indeed, in *Asterias rubens*, proteins related to both production and release of coelomocytes were found at very low levels in non-regenerating conditions (Holm et al. 2008; Hernroth et al. 2010), thus explaining their absence in the herein detected CE proteome. Nonetheless, adducin, identified in the present paper and in *A. rubens*, is a family of membrane skeleton proteins that includes an isoform reported as abundant in mammal hematopoietic tissues (Kiang and Leung 2018). Several other cytoskeletal proteins involved in maintaining plasma membrane integrity, related to regulation and rearrangement of the cytoskeleton, membrane trafficking, and cell motility were identified in *M. glacialis* CE (Table 1), and in coelomocytes of the same species (Franco et al. 2011). Together, the cellular morphological similarities and the several shared proteins between *M. glacialis* coelomocytes and the CE suggest the origin of the former from the latter. This hypothesis is corroborated by the identification of proteins associated with cell motility that may indicate a migration of cells from CE to the coelomic cavity.

Cell adhesion by direct cell interaction (adherent junctions) or through the secreted extracellular matrix (focal adhesion) is critical to epithelial cell polarity, morphogenesis, and mechanosensation. Several proteins involved in these processes were also identified in this work (Table 1), by Gabre et al. (2015) and, interestingly, in coelomocytes (Franco et al. 2011) despite being characteristic of epithelial tissues. Talin interacts with membrane integrins in these indirect cellular junctions that also include the lipoma-preferred partner protein, and both junction types contain the actin-binding protein vinculin (Miller et al. 2018). Catenin and actinin alpha 1 are other components of the adherent junction protein complexes (Yamada et al. 2005). Both adhesion complexes can be involved in signal transduction and regulate tissue development and cell migration. This process is the umbrella for the cell mobility protein cluster that includes several myosins and activating GTPases.

### CE as source of regeneration-competent cells

Echinoderm CE has been often proposed as a source of regeneration-competent cells, i.e., the cellular building-blocks directly responsible for the actual regeneration phenomenon through differentiation processes (Candia Carnevali et al. 2009; Candia Carnevali and Burighel 2010; Hernroth et al. 2010; García-Arrarás et al. 2011; Ben Khadra et al. 2018). This is a clearly distinct CE function from the production of free-wandering coelomocytes, which are specialized in immunological activities. As for the production of regeneration-competent cells, our results suggest that at the level of the regenerating tip (in the area of maximum curvature), coelothelial cells undergo a partial dedifferentiation (i.e., rather high nucleus/cytoplasm ratio), and basally “detach” from the epithelial layer migrating toward the underlying mesenchymal tissue, as indicated by their elongated shapes. This phenomenon perfectly fits with what has been described in other echinoderms. Indeed, in ophiuroids, Piovani and co-workers (Piovani et al. 2021) showed that CE cells at the regenerating arm tip undergo partial dedifferentiation and subsequent epithelial-mesenchymal transition (EMT) to originate sclerocyte precursors. Similarly, in holothuroids, EMT of mesothelial cells in the underlying connective tissue was identified as a key process in gut regeneration (García-Arrarás et al. 2011). In crinoids, Candia Carnevali and Bonasoro (2001) suggested that, in arm regeneration, CE-derived cells contribute to the regeneration of both coelomic cavity itself and coelom-associated arm structures. The constant production of new cells from CE is guaranteed by an intense proliferation of the (de-differentiated) CE cells themselves, as observed in both asteroids and crinoids regenerating arm tips (Candia Carnevali et al. 1995a, 1997; Moss et al. 1998). Overall, this strongly indicates the existence of a phylum-shared mechanism for the production of regeneration-competent cells relying on de-differentiation, proliferation, and subsequent EMT of coelothelial cells (Ben Khadra et al. 2018). In non-regenerating samples, we never observed presumptive stem cells within the CE, although the possibility that these are located in other non-investigated part of the CE cannot be excluded. Our parallel proteomic analysis supported this, as in non-regenerating samples no proteins specific to stem/pluripotent cell production were detected. Once more, these integrated data suggest that echinoderm regeneration relies on ad hoc de-differentiation/migration mechanisms rather than on the use of stocked stem cells.

### Apocrine secretion: mechanical cue and release of immune factors?

One of the most interesting phenomena observed in our experiments was the intense apocrine secretion at the level of the coelothelial cells. In CE cultures from *A. rubens*, Sharlaimova and co-workers (Sharlaimova et al. 2010) observed the production of roundish bodies of about 2-µm diameter (described as “anuclear cells”), similar to those described in the present work, which could also be apocrine secretion products or the result of the in vitro conditions. Likewise, in the same starfish species, Beijnink and Voogt (1986) described the formation of PAS-positive and glycogen-containing apical bulbs from myoepithelial cells of the haemal tuff. Interestingly, the molecular partners of these events are included in the cluster of identified proteins involved in membrane trafficking processes (Table 1) which require that the regulation and rearrangement of the cytoskeleton come into play. In fact, exo- and endocytosis work as coupled processes allowing the maintenance of the vesicle pool while preserving plasma membrane functionalities (Liang et al. 2017). The identification of clathrin suggests the presence of the clathrin-mediated endocytic pathway in CE cells. The rearrangement of the cytoskeleton involves also the regulation of the aforementioned pathway by the Rho family of small GTPase, a group of key regulatory molecules that link surface receptors to the actin cytoskeleton (Goitre et al. 2014).

Our observations indicate that this secretion activity is particularly evident in the distal-most tip of both non-regenerating and regenerating coelomic cavities. A similar phenomenon of apocrine secretion was observed also during arm tip regeneration in other echinoderm species, including crinoids (Candia Carnevali et al. 1993). This strongly suggests another phylum-shared mechanism occurring during arm growth/regrowth. A speculative hypothesis is that the intense fluid secretion by the CE could contribute to create a high local hydrostatic pressure mechanically pushing the apical regrowth and driving the regeneration process (Ben Khadra et al. 2018). Indeed, this is in line with what is observed during the development of many model organisms, where tubulogenesis and axis formation are partially driven by “fluid forces” generated by fluid accumulation within the lumina (Olver et al. 2004; Navis and Bagnat 2015; Chan and Hiiragi 2020). This increased internal pressure is generally due to an intense secretion of the epithelium of the organ itself (Tsarouhas et al. 2007). More specific analytical methods are however necessary to confirm the fascinating hypothesis of a hydrostatic-driven arm regeneration process in echinoderms.

A further hypothesis is that the secreted vesicles contain regulatory molecules, particularly related to the immune response. Indeed, in echinoderms, the humoral immune response is mediated by a wide variety of secreted molecules found in the coelomic fluid: they include proteins, like lectins, essential for recognizing, neutralizing or destroying foreign material by promoting cell migration, agglutination, and phagocytosis (Franco et al. 2011; Dheilly et al. 2013), or other immune-active molecules possessing antibacterial and antiviral activities, including saponins (Laires 2012). Several proteins found in *M. glacialis* CE proteome are proper of the immune response (Table 1). The scavenger receptor, known to act as a phagocytic receptor, was also detected in sea urchin coelomocytes (Smith et al. 2010). Macrophages expressing this receptor have also been found in mammals during the healing phase of acute inflammation, in chronic inflammation, and in wound healing tissue (Fabriek et al. 2005). Another interesting protein is the gp96 endoplasmic reticulum chaperone for cell-surface Toll-like receptors (TLRs). These membrane receptors, important for innate immunity, are expressed in macrophages and recognize lipopolysaccharide (LPS), a ubiquitous pathogen membrane molecule (Yang et al. 2007). Gene expression of Ras-related protein Rab, a protein from the small GTPases Rab family, is induced by LPS to regulate the replenishing of the plasma membrane with TLRs (Yeo et al. 2016). The Rab GDP dissociation inhibitor regulates the GDP/GTP exchange reaction by inhibiting the dissociation of GDP from them, and the subsequent binding of GTP. TLRs are also expressed in *Strongylocentrotus purpuratus* coelomocytes (Smith et al. 2010), underlining the immune function of these cells. Evidence of CE phagocytic function is also given by the identification of proteins of the Arp2/3 complex. The Arp2/3 is a complex involved in the regulation of the actin cytoskeleton and in the formation of protrusive processes, like pseudopodia and filopodia, that is a crucial event for phagocytosis, a central process in immunity and, most of all, in autophagy and debris clearance (May et al. 2000). The Rho-related protein (RacB) and cell division cycle 42 (Cdc42) protein, both found in our analyses, are required for the activation of the Arp2/3 complex to form protrusive structures during phagocytosis, whereas Rho controls actin assembly at phagosomes (Olazabal et al. 2002).

Furthermore, the mitogen-activated protein kinase 14 (p38-α), also identified in this study, is a tyrosine-phosphorylated protein usually detected in activated macrophages that has an essential role in the induction of tumor necrosis factor-α (TNF-α) in mammals (Han et al. 1994). TNF-α is usually expressed in mammals, mainly by macrophages and epidermal cells (Pastar et al. 2014), and has a key regulatory function in the inflammatory response (Bradley 2008). It is also involved in the activation, migration, and differentiation of keratinocytes and fibroblast activation after injury (Pastar et al. 2014). The identification of p38-α in *M. glacialis* CE proteome might be also considered an indirect evidence of the expression of TNF-α. A gene homologous to the mammalian TNF-α has been described in echinoderms (Matranga et al. 2005), namely in the sea urchin *Strongylocentrotus purpuratus*, the starfish *Patiria miniata* (Cameron et al. 2009), and the brittle star *Amphiura filiformis* (Dylus et al. 2018). In echinoderms, this protein is expressed in coelomocytes following physical stress (Ben Khadra et al. 2017). Interestingly, the presence of TNF-α in normal conditions has also been described in the coelomic fluid of *Asterias forbesi* (Beck and Habicht 1986), suggesting a possible secretion of this protein from the CE into the coelomic cavity. Thus, these results suggest that CE could be constantly involved in a basal inflammatory response.

From the proteins listed in Table 1, 43% are classified by KEGG as exosomal proteins. Exosomes are generated through an endosomal pathway where multivesicular bodies fuse with the plasma membrane. These membrane vesicles (30–150 nm in size) offer a potent mechanism for communication between nearby or distant cells or tissues in order to change their physiological functions and properties in specific innate and adaptive immune responses, as reported in several marine invertebrates. Additionally, in the Eastern oyster *Crassostrea virginica*, exosome-like vesicles relating to shell formation and repair have been observed (Auguste et al. 2020).

In light of these results, there is a strong hypothesis that CE has exo/endocytic functions which are involved in the constant growth and regeneration of the arm tip such as immune response against pathogens, autophagy, and debris clearance (Hyman 1955).

### Clearing, “recycling,” and protein synthesis functions

As previously described, the histological organization of *M. glacialis* CE is similar to that of other starfish: it is a typical myo-epithelium, which, besides apical peritoneocytes, includes two layers of myocytes, longitudinally and transversally oriented (Hyman 1955; García-Arrarás and Dolmatov 2010; Ben Khadra et al. 2015). Interestingly, in our samples, a sub-layer of peculiar flagellated and vesicular cells, never described before, has been detected in the inner borders of the circular muscle layer. Two identified proteins specifically associated to ciliary motility, also detected in *C. muricata* CE (Gabre et al. 2015), corroborate the presence of ciliated cells. Axonemal dynein is the molecular motor that drives the beating of cilia and flagella regulated by the radial spokes (radial spoke head protein 9). The origin of the observed vesicular layer is uncertain. From a histological point of view, these vesicular cells belong to the circular muscle layer itself and can therefore be considered part of the myo-epithelial layer, as also suggested by the presence of scattered myocyte among them. These cells showed two distinctive features which can be indicative of their putative functions and deserve to be discussed separately as follows: (1) They display a remarkable phagocytic activity that is particularly localized in the tip of non-regenerating arms (an area of ongoing remodeling and growth) but much more evident in regenerating arms. This clearing activity increases in parallel with the disappearance of CE muscle layers and their phagosomes often contain the contractile apparatus of degenerating myocytes. This clearly indicates a direct involvement of this particular cell layer in remodeling stump structures, specially muscle cells. During echinoderm regeneration events, remarkable myocyte disorganization and dedifferentiation phenomena have been widely documented (Bonasoro et al. 1998; Candia Carnevali and Bonasoro 2001; Dolmatov and Ginanova 2001; García-Arrarás and Dolmatov 2010; García-Arrarás et al. 2011; Ben Khadra et al. 2015, 2017). At present, it is still an open question if de-differentiated/partially digested myocytes become a source of regeneration-competent multipotent cells, or if they are completely recycled (through phagocytosis) and used as a source of material/energy for the regenerative process, or if both pathways can occur (Candia Carnevali and Bonasoro 2001; Ben Khadra et al. 2015, 2017). In this study, we never observed putative undifferentiated cells in the vesicular cell layer: this leads us to suppose that the myocyte rearrangement is mainly functional to recover and recycle materials for the constant growth/regrowth of the arm. (2) The vesicular cells display an abundant cytoplasm, with massive presence of swollen endoplasmic reticulum (ER) cisternae and Golgi apparatuses (one or more), and a euchromatic nucleus. These ultrastructural features suggest an intense protein synthesis activity, particularly related to glycoproteins. The protein turnover process is extensively represented within the identified proteins in *M. glacialis* CE, including the ubiquitin proteasome system and protein processing in the ER and Golgi apparatus (Table 1). The vesicles in those cells could be autophagosomes which are structures of the intracellular degradation system for cytoplasmic contents (macroautophagy system), or Golgi vesicles. Golgi vesicles are directly originated from the Golgi, where proteins received from the ER are further processed and sorted for transport to lysosomes, plasma membrane, or secretion.

Several proteins, such as calreticulin, are associated with both autophagy and the immune response. Calreticulin is a multifunctional Ca^2+^-binding protein that regulates intracellular Ca^2+^ homeostasis and its storage in the ER, and whose abundance increases in response to bacterial challenges (Zhang et al. 2014). Several chaperons, like endoplasmic reticulum chaperone BiP, are members of the endoplasmic reticulum quality-control system tasked with detecting proteins that fail to mature properly and to target them for cytosolic degradation (Pobre et al. 2019). Otherwise, misfolded or partially unfolded proteins are recognized by the GroE chaperonin system that directs them to continue the folding process (O’Neil et al. 2018). Other factors intervening in the folding process are the FK506 binding proteins (FKBPs) and cyclophilin B, members of a large family of proteins that possess peptidyl prolyl cis/trans isomerase (PPIase) domains, which alter the conformation of target proteins (Tong and Jiang 2015; Khong et al. 2020). The vesicle-associated membrane protein–associated protein A is localized in the ER/Golgi pathway. The interaction with other components of the vesicle trafficking machinery as well as the similar topology and coiled-coil structure to the t-SNARE syntaxin family suggest a role in vesicle transport (Wyles et al. 2002). Sorting nexins belong to a family of peripheral proteins that contain a phosphoinositide-binding domain. This lipid is a component of cell organelles that regulates a variety of sorting processes ensuring the proper distribution of endocytic cargo that is trafficked through the endosome for degradation or returned to the plasma membrane after recycling (Naslavsky and Caplan 2018). Another cell degradation system is the ubiquitin–proteasome where, upon ubiquitination, proteins become degraded by the proteasome (a complex of proteases) (Kleiger and Mayor 2014).

## Conclusions

Overall, the present study demonstrated that starfish CE is not simply a “container” of the coelomic fluid, defining and delimiting the coelomic environment from the surrounding body tissues. Indeed, both ultrastructural and proteomic investigations suggest that it is a highly complex tissue that plays diverse functions and is involved in several key physiological processes. In fact, in both normal homeostatic and regeneration conditions, it provides both cells and molecules (and possibly also mechanical devices) indispensable for the fulfillment of different physiological functions. This view is perfectly in line with its “organogenetic” role during the development of many coelomate animals (Ariza et al. 2016).

Nevertheless, further specific molecular investigations are needed, in particular addressed to understand which molecules are actively synthesized by the unusual vesicular cell population observed in this study. This should help to elucidate the putative role of the CE in the immune system and/or its involvement in the production/release of fundamental molecules involved in both homeostatic and regenerative processes, such as cytokines TGF-β and TNF-α.

Our combined approaches of microscopic anatomy and proteomics appear to have partially shed light on CE roles in the physiology of the starfish *M. glacialis*, setting solid basis for future more in-depth investigations which can provide a complete overview of the morpho-functional adaptive modifications and evolution of this tissue among coelomates.

## Supplementary Information

Below is the link to the electronic supplementary material.

Supplementary file1 Online Resource 1 SWATH-MS method m/z windows. (DOCX 18 KB)

Supplementary file2 Online Resource 2 CE of the stump in a 48 p.a. sample. Apocrine secretion (arrow) from the peritoneocytes (p) is still visible. Abbreviations: pc: perivisceral coelom; lm: longitudinal muscle layer; ct: connective tissue; cm: circular muscle layer; d: dermis. (TIF 9956 KB)

Supplementary file3 Online Resource 3 Identified proteins in each biological replicate (A1E1, B1F1 and C1D1) using Information-Dependent Acquisition (IDA) and Sequential Window Acquisition of All Theoretical Mass Spectra (SWATH-MS) methods. (XLSX 62 KB)

Supplementary file4 Online Resource 4 Combined list of identified proteins and their molecular functions. (XLSX 61 KB)

Supplementary file5 Online Resource 5 Full annotation of the identified proteins using the UniProt Gene Ontology information by STRAP analysis. (XLSX 86 KB)

Supplementary file5 Online Resource 6 STRING analysis of the coelomic epithelium identified proteins. The functions attributed to each cluster are A), C), H) membrane trafficking, B) cell motility, D) cell-cell adhesion, E), G) phagocytosis, F) protein processing in the endoplastic reticulum and export, I) ciliary motility, J) methionine degradation, K) ubiquitin proteosome system and L) plasma membrane integrity. (JPG 301 KB)

## Declarations

### Research involving animals

The present research was performed according to the national laws; the species used under this study are not endangered and their use does not require any ethical approval.

## References

[CR1] Andrade C, Oliveira B, Guatelli S (2021). Characterization of coelomic fluid cell types in the starfish Marthasterias glacialis using a flow cytometry/imaging combined approach. Front Immunol.

[CR2] Anjo SI, Santa C, Manadas B (2015). Short GeLC-SWATH: a fast and reliable quantitative approach for proteomic screenings. Proteomics.

[CR3] Ariza L, Carmona R, Cañete A (2016). Coelomic epithelium-derived cells in visceral morphogenesis. Dev Dyn.

[CR4] Auguste M, Balbi T, Ciacci C, Canesi L (2020). Conservation of cell communication systems in invertebrate host–defence mechanisms: possible role in immunity and disease. Biology.

[CR5] Beck G, Habicht GS (1986). Isolation and characterization of a primitive interleukin-1-like protein from an invertebrate, Asterias forbesi. Proc Natl Acad Sci U S A.

[CR6] Beijnink FB, Voogt PA (1986). The aboral haemal system of the sea star, Asterias rubens (Echinodermata, Asteroidea): an ultrastructural and histochemical study. Zoomorphology.

[CR7] Ben Khadra Y, Ferrario C, Di Benedetto C (2015). Wound repair during arm regeneration in the red starfish Echinaster sepositus. Wound Repair Regen.

[CR8] Ben Khadra Y, Sugni M, Ferrario C (2018). Regeneration in stellate echinoderms: Crinoidea, Asteroidea and Ophiuroidea. Results Probl Cell Differ.

[CR9] Ben Khadra Y, Sugni M, Ferrario C (2017). An integrated view of asteroid regeneration: tissues, cells and molecules. Cell Tissue Res.

[CR10] Bhatia VN, Perlman DH, Costello CE, McComb ME (2009). Software tool for researching annotations of proteins: open-source protein annotation software with data visualization. Anal Chem.

[CR11] Biressi ACM, Zou T, Dupont S (2010). Wound healing and arm regeneration in Ophioderma longicaudum and Amphiura filiformis (Ophiuroidea, Echinodermata): comparative morphogenesis and histogenesis. Zoomorphology.

[CR12] Bonasoro F, Carnevali C, Moss C, Thorndyke M (1998) Epimorphic versus morphallactic mechanism in arm regeneration of crinoids and asteroids: pattern of cell proliferation/differentiation and cell lineage

[CR13] Bossche JPV, Jangoux M (1976). Epithelial origin of starfish coelomocytes. Nature.

[CR14] Bradley JR (2008). TNF-mediated inflammatory disease. J Pathol.

[CR15] Butt RH, Coorssen JR (2006). Pre-extraction sample handling by automated frozen disruption significantly improves subsequent proteomic analyses. J Proteome Res.

[CR16] Cameron RA, Samanta M, Yuan A (2009). SpBase: the sea urchin genome database and web site. Nucleic Acids Res.

[CR17] Candia Carnevali MD (2006) Regeneration in echinoderms: repair, regrowth, cloning. Invertebr Surviv J 3

[CR18] Candia Carnevali MD, Bonasoro F (2001). Microscopic overview of crinoid regeneration. Microsc Res Tech.

[CR19] Candia Carnevali MD, Bonasoro F, Biale A (1997). Pattern of bromodeoxyuridine incorporation in the advanced stages of arm regeneration in the feather star Antedon mediterranea. Cell Tissue Res.

[CR20] Candia Carnevali MD, Bonasoro F, Lucca E, Thorndyke MC (1995). Pattern of cell proliferation in the early stages of arm regeneration in the feather star Antedon mediterranea. J Exp Zool.

[CR21] Candia Carnevali MD, Burighel P (2010) Regeneration in echinoderms and ascidians. In: eLS. Am Cancer Soc

[CR22] Candia Carnevali MD, Lucca E, Bonasoro F (1993). Mechanisms of arm regeneration in the feather star Antedon mediterranea: healing of wound and early stages of development. J Exp Zool.

[CR23] Candia Carnevali MD, Thorndyke MC, Matranga V, Rinkevich B, Matranga V (2009). Regenerating echinoderms: a promise to understand stem cells potential. Stem Cells in Marine Organisms.

[CR24] Chan CJ, Hiiragi T (2020). Integration of luminal pressure and signalling in tissue self-organization. Development.

[CR25] Candia Carnevali MD, Bonasoro F, Wilkie IC (1995b) Coelom and “tinkering”in echinoids: morphofunctional adaptations of the lantern coelom. In: Lanzavecchia G, Valvassori R, Candia Carnevali MD (eds) Body cavities: function and phylogeny. Mucchi, Modena, pp 135–165

[CR26] Dheilly NM, Raftos DA, Haynes PA (2013). Shotgun proteomics of coelomic fluid from the purple sea urchin, Strongylocentrotus purpuratus. Dev Comp Immunol.

[CR27] Dolmatov IY (1993). Proliferation of tissues of regenerating aquapharyngeal complex in holoturians. Russ J Dev Biol.

[CR28] Dolmatov IY, Ginanova TT (2001). Muscle regeneration in holothurians. Microsc Res Tech.

[CR29] Dolmatov IY, Mashanov VS, Zueva OR (2007). Derivation of muscles of the Aristotle’s lantern from coelomic epithelia. Cell Tissue Res.

[CR30] Dylus DV, Czarkwiani A, Blowes LM (2018). Developmental transcriptomics of the brittle star Amphiura filiformis reveals gene regulatory network rewiring in echinoderm larval skeleton evolution. Genome Biol.

[CR31] Fabriek BO, Dijkstra CD, van den Berg TK (2005). The macrophage scavenger receptor CD163. Immunobiology.

[CR32] Fasoli E, D’Amato A, Kravchuk AV (2011). Popeye strikes again: the deep proteome of spinach leaves. J Proteomics.

[CR33] Ferrario C, Ben Khadra Y, Czarkwiani A (2018). Fundamental aspects of arm repair phase in two echinoderm models. Dev Biol.

[CR34] Franco CF, Santos R, Coelho AV (2011). Proteome characterization of sea star coelomocytes–the innate immune effector cells of echinoderms. Proteomics.

[CR35] Gabre JL, Martinez P, Nilsson Sköld H (2015). The coelomic epithelium transcriptome from a clonal sea star, Coscinasterias muricata. Mar Genomics.

[CR36] García-Arrarás JE, Dolmatov IYu (2010). Echinoderms; potential model systems for studies on muscle regeneration. Curr Pharm Des.

[CR37] García-Arrarás JE, Valentín-Tirado G, Flores JE et al (2011) Cell dedifferentiation and epithelial to mesenchymal transitions during intestinal regeneration in H. glaberrima. BMC Dev Biol 11:61. 10.1186/1471-213X-11-61 10.1186/1471-213X-11-61 PMC3207902 22004330

[CR38] Gillet LC, Navarro P, Tate S (2012). Targeted data extraction of the MS/MS spectra generated by data-independent acquisition: a new concept for consistent and accurate proteome analysis. Mol Cell Proteomics.

[CR39] Ginanova TT (2007). Participation of the coelomic epithelium of the body wall in muscle regeneration in Apostichopus japonicus (Holothuroidea: Aspidochirota). Russ J Mar Biol.

[CR40] Goitre L, Trapani E, Trabalzini L, Retta SF (2014). The Ras superfamily of small GTPases: the unlocked secrets. Methods Mol Biol.

[CR41] Golconda P, Buckley KM, Reynolds CR (2019). The axial organ and the pharynx are sites of hematopoiesis in the sea urchin. Front Immunol.

[CR42] Gomes LP, Anjo SI, Manadas B (2019). Proteomic analyses reveal new insights on the antimicrobial mechanisms of chitosan biopolymers and their nanosized particles against Escherichia coli. Int J Mol Sci.

[CR43] Gorshkov AN, Blinova MI, Pinaev GP (2009) Ultrastructure of coelomic epithelium and coelomocytes of the starfish Asterias rubens L. in norm and after wounding. Cell Tiss Biol 3:477. 10.1134/S1990519X09050113 19799349

[CR44] Gros J, Tabin CJ (2014). Vertebrate limb bud formation is initiated by localized epithelial to mesenchymal transition. Science.

[CR45] Han J, Lee JD, Bibbs L, Ulevitch RJ (1994). A MAP kinase targeted by endotoxin and hyperosmolarity in mammalian cells. Science.

[CR46] Hernroth B, Farahani F, Brunborg G (2010). Possibility of mixed progenitor cells in sea star arm regeneration. J Exp Zool B Mol Dev Evol.

[CR47] Holm K, Dupont S, Sköld H (2008). Induced cell proliferation in putative haematopoietic tissues of the sea star, Asterias rubens (L.). J Exp Biol.

[CR48] Hyman LH (1955). The invertebrates. Vol.

[CR49] Kaneshiro ES, Karp RD (1980). The ultrastructure of coelomocytes of the sea star dermasterias imbricata. Biol Bull.

[CR50] Khong ML, Li L, Solesio ME (2020). Inorganic polyphosphate controls cyclophilin B-mediated collagen folding in osteoblast-like cells. FEBS J.

[CR51] Kiang KM-Y, Leung GK-K (2018). A review on adducin from functional to pathological mechanisms: future direction in cancer. Biomed Res Int.

[CR52] Kim C-H, Go H-J, Oh HY (2018). Transcriptomics reveals tissue/organ-specific differences in gene expression in the starfish Patiria pectinifera. Mar Genomics.

[CR53] Kleiger G, Mayor T (2014). Perilous journey: a tour of the ubiquitin-proteasome system. Trends Cell Biol.

[CR54] Kozlova AB, Petukhova OA, Pinaev GP (2006). The analysis of cellular elements in coelomic fluid during early regeneration of of the starfish Asterias rubens L. Tsitologiia.

[CR55] Laires R (2012) Characterization of the coelomic fluid of the starfish Marthasterias glacialis in a wound-healing phase. Universitade Tecnica de Lisboa

[CR56] Liang K, Wei L, Chen L (2017). Exocytosis, endocytosis, and their coupling in excitable cells. Front Mol Neurosci.

[CR57] Matranga V, Pinsino A, Celi M (2005). Monitoring chemical and physical stress using sea urchin immune cells. Prog Mol Subcell Biol.

[CR58] May RC, Caron E, Hall A, Machesky LM (2000). Involvement of the Arp2/3 complex in phagocytosis mediated by FcgammaR or CR3. Nat Cell Biol.

[CR59] Miller PW, Pokutta S, Mitchell JM (2018). Analysis of a vinculin homolog in a sponge (phylum Porifera) reveals that vertebrate-like cell adhesions emerged early in animal evolution. J Biol Chem.

[CR60] Moss C, Hunter AJ, Thorndyke MC (1998) Patterns of bromodeoxyuridine incorporation and neuropeptide immunoreactivity during arm regeneration in the star^®^sh Asterias rubens. 16

[CR61] Muñoz-Chápuli R, Carmona R, Guadix JA (2005). The origin of the endothelial cells: an evo-devo approach for the invertebrate/vertebrate transition of the circulatory system. Evol Dev.

[CR62] Mutsaers SE, Wilkosz S (2007). Structure and function of mesothelial cells. Cancer Treat Res.

[CR63] Naslavsky N, Caplan S (2018) The enigmatic endosome - sorting the ins and outs of endocytic trafficking. J Cell Sci 131:jcs216499. 10.1242/jcs.216499 10.1242/jcs.216499 PMC6051342 29980602

[CR64] Navis A, Bagnat M (2015). Developing pressures: fluid forces driving morphogenesis. Curr Opin Genet Dev.

[CR65] Olazabal IM, Caron E, May RC (2002). Rho-kinase and myosin-II control phagocytic cup formation during CR, but not FcgammaR, phagocytosis. Curr Biol.

[CR66] Olver RE, Walters DV, Wilson M, S,  (2004). Developmental regulation of lung liquid transport. Annu Rev Physiol.

[CR67] O’Neil PT, Machen AJ, Deatherage BC (2018). The Chaperonin GroEL: a versatile tool for applied biotechnology platforms. Front Mol Biosci.

[CR68] Pastar I, Stojadinovic O, Yin NC (2014). Epithelialization in wound healing: a comprehensive review. Adv Wound Care (new Rochelle).

[CR69] Pinsino A, Thorndyke MC, Matranga V (2007). Coelomocytes and post-traumatic response in the common sea star Asterias rubens. Cell Stress Chaperones.

[CR70] Piovani L, Czarkwiani A, Ferrario C (2021). Ultrastructural and molecular analysis of the origin and differentiation of cells mediating brittle star skeletal regeneration. BMC Biol.

[CR71] Pobre KFR, Poet GJ, Hendershot LM (2019). The endoplasmic reticulum (ER) chaperone BiP is a master regulator of ER functions: getting by with a little help from ERdj friends. J Biol Chem.

[CR72] Rieger RM, Lombardi J (1987). Ultrastructure of coelomic lining in echinoderm podia: significance for concepts in the evolution of muscle and peritoneal cells. Zoomorphology.

[CR73] Sennels L, Bukowski-Wills J-C, Rappsilber J (2009). Improved results in proteomics by use of local and peptide-class specific false discovery rates. BMC Bioinformatics.

[CR74] Sharlaimova N, Pinaev G, Petukhova O (2010). Comparative analysis of behavior and proliferative activity in culture of cells of coelomic fluid and of cells of various tissues of the sea star Asterias rubens L. Isolated from normal and injured animals. Cell and Tissue Biology.

[CR75] Sharlaimova N, Shabelnikov S, Bobkov D (2021). Coelomocyte replenishment in adult Asterias rubens: the possible ways. Cell Tissue Res.

[CR76] Sharlaimova N, Shabelnikov S, Petukhova O (2014). Small coelomic epithelial cells of the starfish Asterias rubens L. that are able to proliferate in vivo and in vitro. Cell Tissue Res.

[CR77] Sharlaimova NS, Petukhova OA (2012). Characteristics of populations of the coelomic fluid and coelomic epithelium cells from the starfish Asterias rubens L. able attach to and spread on various substrates. Cell Tiss Biol.

[CR78] Smith LC, Ghosh J, Buckley KM (2010). Echinoderm immunity. Adv Exp Med Biol.

[CR79] Szklarczyk D, Franceschini A, Wyder S (2015). STRING v10: protein-protein interaction networks, integrated over the tree of life. Nucleic Acids Res.

[CR80] Teixeira FG, Carvalho MM, Panchalingam KM (2017). Impact of the secretome of human mesenchymal stem cells on brain structure and animal behavior in a rat model of Parkinson’s disease. Stem Cells Transl Med.

[CR81] Tong M, Jiang Y (2015). FK506-binding proteins and their diverse functions. Curr Mol Pharmacol.

[CR82] Tsarouhas V, Senti K-A, Jayaram SA (2007). Sequential pulses of apical epithelial secretion and endocytosis drive airway maturation in Drosophila. Dev Cell.

[CR83] Wood RL, Cavey MJ (1981). Ultrastructure of the coelomic lining in the podium of the starfish Stylasterias forreri. Cell Tissue Res.

[CR84] Wyles JP, McMaster CR, Ridgway ND (2002). Vesicle-associated membrane protein-associated protein-A (VAP-A) interacts with the oxysterol-binding protein to modify export from the endoplasmic reticulum. J Biol Chem.

[CR85] Yamada S, Pokutta S, Drees F (2005). Deconstructing the cadherin-catenin-actin complex. Cell.

[CR86] Yang Y, Liu B, Dai J (2007). Heat shock protein gp96 is a master chaperone for toll-like receptors and is important in the innate function of macrophages. Immunity.

[CR87] Yeo JC, Wall AA, Luo L, Stow JL (2016). Sequential recruitment of Rab GTPases during early stages of phagocytosis. Cell Logist.

[CR88] Zhang P, Li C, Li Y (2014). Proteomic identification of differentially expressed proteins in sea cucumber Apostichopus japonicus coelomocytes after Vibrio splendidus infection. Dev Comp Immunol.

