# Supplementary material for: More than a simple epithelial layer: multifunctional role of echinoderm coelomic epithelium
Source: Cell Tissue Res. 2022 Sep 9;390(2):207–27. doi: 10.1007/s00441-022-03678-x (PMC9630195; doi:10.1007/s00441-022-03678-x)
Supplement: Supplementary file 1 — Supplementary file1 Online Resource 1 SWATH-MS method m/z windows. (DOCX 18 KB) [file 441_2022_3678_MOESM1_ESM.docx]

*More than a simple epithelial layer: multifunctional role of echinoderm coelomic epithelium,Cell and Tissue Research, Silvia Guatelli1, Cinzia Ferrario1, 2, Francesco Bonasoro1, Sandra I. Anjo3, Bruno Manadas3, Maria Daniela Candia Carnevali1, Ana Varela Coelho*4, Michela Sugni*1, 2; Corresponding authors: michela.sugni@unimi.it, Department of Environmental Science and Policy-University of Milan, Via Celoria 26, 20133 Milan, Italy; varela@itqb.unl.pt, ITQB-Instituto de Tecnologia Química e Biológica António Xavier, Universidade Nova de Lisboa, Av. da República, 2780-157 Oeiras, Portugal*

Online Resource 1- SWATH-MS method *m/z* windows

|  | ***m/z* range** | **Width (Da)** | **Collision Energy Spread (CES)** |
| --- | --- | --- | --- |
| **Window 1** | 349.5-360.9 | 11.4 | 5 |
| **Window 2** | 359.9-375.2 | 15.3 | 5 |
| **Window 3** | 374.2-389.2 | 15 | 5 |
| **Window 4** | 388.2-402.2 | 14 | 5 |
| **Window 5** | 401.2-415.3 | 14.1 | 5 |
| **Window 6** | 414.3-427.4 | 13.1 | 5 |
| **Window 7** | 426.4-439.1 | 12.7 | 5 |
| **Window 8** | 438.1-449.9 | 11.8 | 5 |
| **Window 9** | 448.9-460.7 | 11.8 | 5 |
| **Window 10** | 459.7-471.1 | 11.4 | 5 |
| **Window 11** | 470.1-480.5 | 10.4 | 5 |
| **Window 12** | 479.5-490 | 10.5 | 5 |
| **Window 13** | 489-499 | 10 | 5 |
| **Window 14** | 498-508 | 10 | 5 |
| **Window 15** | 507-516.5 | 9.5 | 5 |
| **Window 16** | 515.5-525.1 | 9.6 | 5 |
| **Window 17** | 524.1-533.2 | 9.1 | 5 |
| **Window 18** | 532.2-540.8 | 8.6 | 5 |
| **Window 19** | 539.8-548.5 | 8.7 | 5 |
| **Window 20** | 547.5-555.7 | 8.2 | 5 |
| **Window 21** | 554.7-563.4 | 8.7 | 5 |
| **Window 22** | 562.4-570.6 | 8.2 | 5 |
| **Window 23** | 569.6-577.8 | 8.2 | 5 |
| **Window 24** | 576.8-585.4 | 8.6 | 5 |
| **Window 25** | 584.4-592.6 | 8.2 | 5 |
| **Window 26** | 591.6-600.3 | 8.7 | 5 |
| **Window 27** | 599.3-607.9 | 8.6 | 5 |
| **Window 28** | 606.9-615.6 | 8.7 | 5 |
| **Window 29** | 614.6-623.2 | 8.6 | 5 |
| **Window 30** | 622.2-630.9 | 8.7 | 5 |
| **Window 31** | 629.9-638.5 | 8.6 | 5 |
| **Window 32** | 637.5-646.2 | 8.7 | 5 |
| **Window 33** | 645.2-653.8 | 8.6 | 5 |
| **Window 34** | 652.8-661.5 | 8.7 | 5 |
| **Window 35** | 660.5-669.1 | 8.6 | 5 |
| **Window 36** | 668.1-677.2 | 9.1 | 5 |
| **Window 37** | 676.2-685.3 | 9.1 | 5 |
| **Window 38** | 684.3-693.9 | 9.6 | 5 |
| **Window 39** | 692.9-702.9 | 10 | 5 |
| **Window 40** | 701.9-711.9 | 10 | 5 |
| **Window 41** | 710.9-721.3 | 10.4 | 5 |
| **Window 42** | 720.3-731.2 | 10.9 | 5 |
| **Window 43** | 730.2-741.6 | 11.4 | 5 |
| **Window 44** | 740.6-752.4 | 11.8 | 5 |
| **Window 45** | 751.4-763.6 | 12.2 | 5 |
| **Window 46** | 762.6-775.8 | 13.2 | 5 |
| **Window 47** | 774.8-787.9 | 13.1 | 5 |
| **Window 48** | 786.9-800.5 | 13.6 | 5 |
| **Window 49** | 799.5-814.5 | 15 | 8 |
| **Window 50** | 813.5-829.3 | 15.8 | 8 |
| **Window 51** | 828.3-845.5 | 17.2 | 8 |
| **Window 52** | 844.5-865.3 | 20.8 | 8 |
| **Window 53** | 864.3-886.5 | 22.2 | 8 |
| **Window 54** | 885.5-911.2 | 25.7 | 8 |
| **Window 55** | 910.2-939.1 | 28.9 | 8 |
| **Window 56** | 938.1-972 | 33.9 | 8 |
| **Window 57** | 971-1008.4 | 37.4 | 10 |
| **Window 58** | 1007.4-1053.4 | 46 | 10 |
| **Window 59** | 1052.4-1120 | 67.6 | 10 |
| **Window 60** | 1119-1249.6 | 130.6 | 10 |
