# Supplementary figures and images for: More than a simple epithelial layer: multifunctional role of echinoderm coelomic epithelium
Source: Cell Tissue Res. 2022 Sep 9;390(2):207–27. doi: 10.1007/s00441-022-03678-x (PMC9630195; doi:10.1007/s00441-022-03678-x)

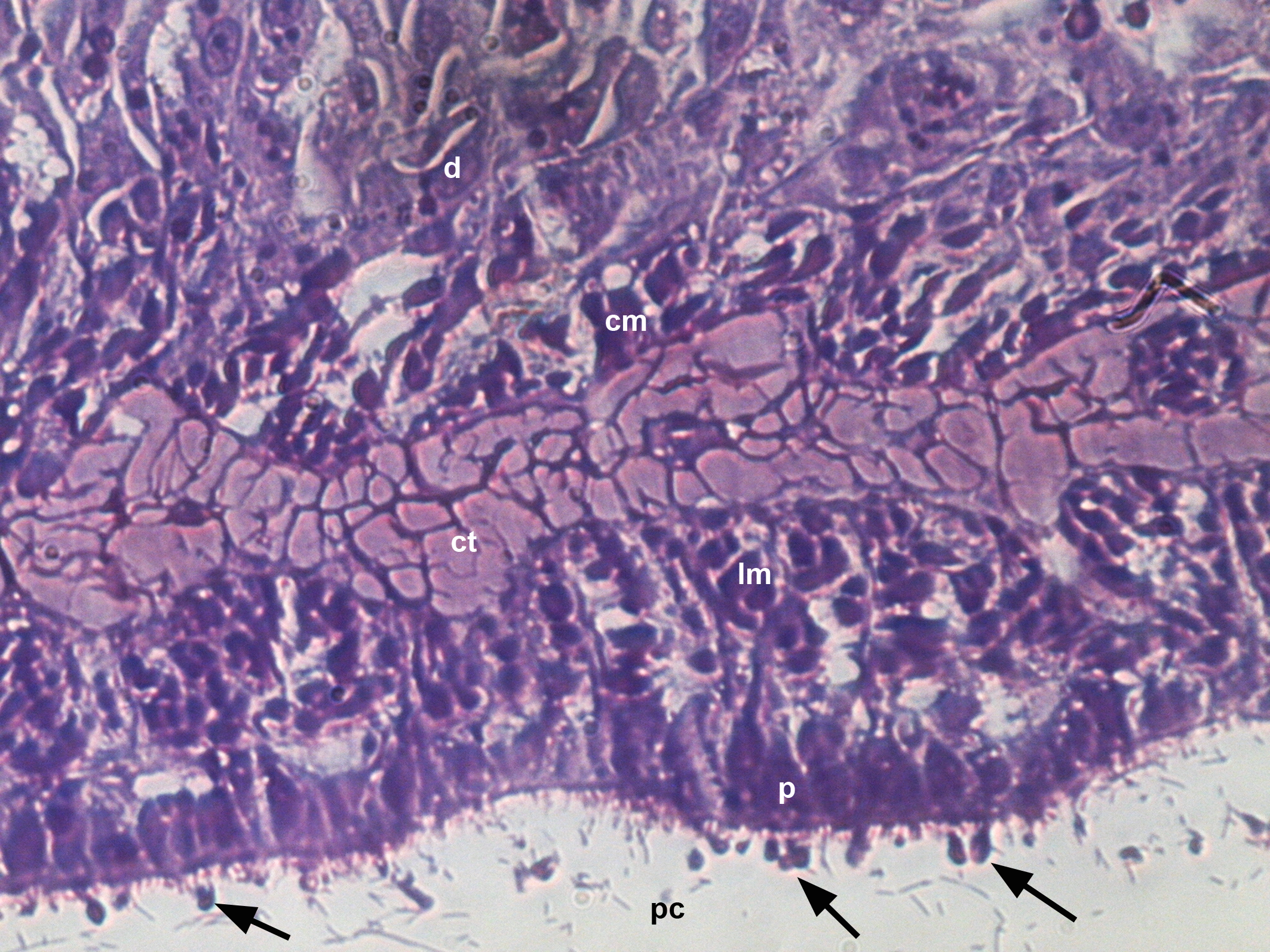

Supplement: Supplementary file 2 — Supplementary file2 Online Resource 2 CE of the stump in a 48 p.a. sample. Apocrine secretion (arrow) from the peritoneocytes (p) is still visible. Abbreviations: pc: perivisceral coelom; lm: longitudinal muscle layer; ct: connective tissue; cm: circular muscle layer; d: dermis. (TIF 9956 KB) [file 441_2022_3678_MOESM2_ESM.tif]

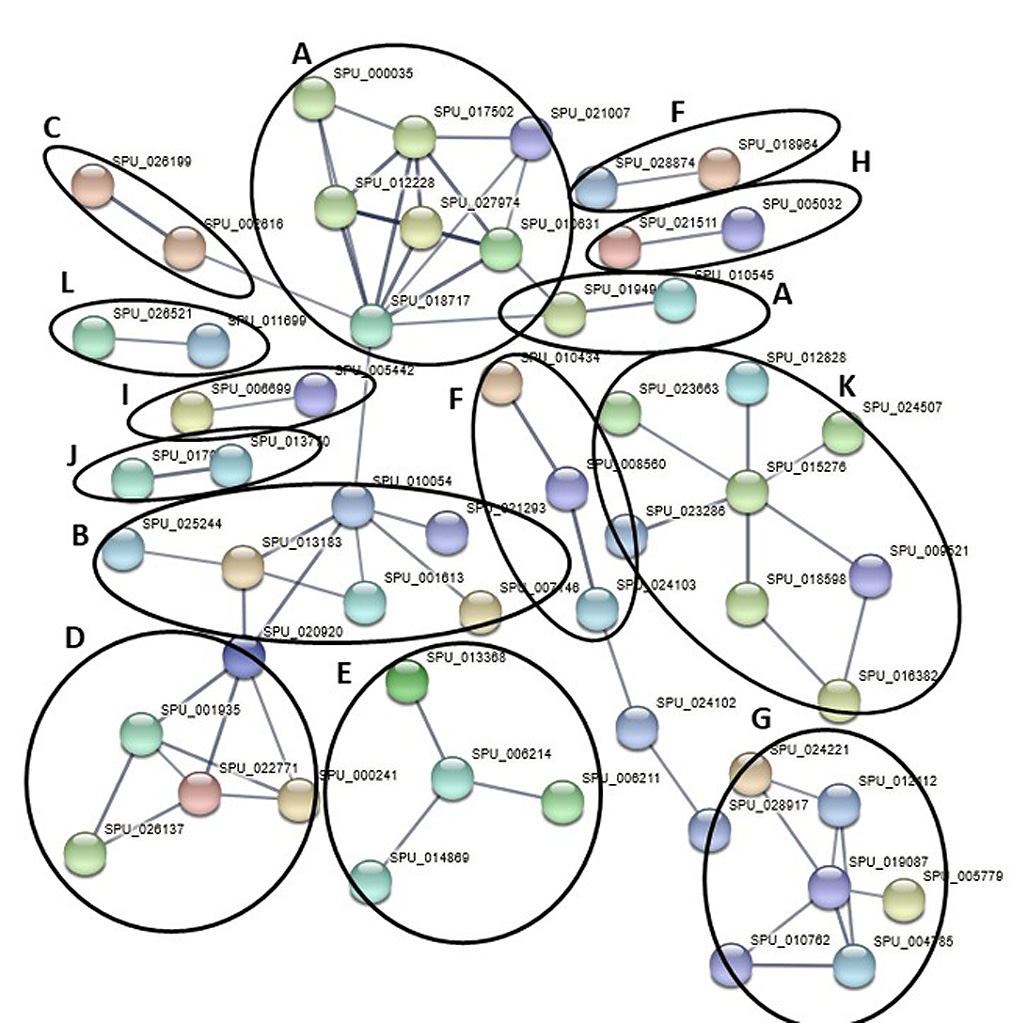

Supplement: Supplementary file 6 — Supplementary file5 Online Resource 6 STRING analysis of the coelomic epithelium identified proteins. The functions attributed to each cluster are A), C), H) membrane trafficking, B) cell motility, D) cell-cell adhesion, E), G) phagocytosis, F) protein processing in the endoplastic reticulum and export, I) ciliary motility, J) methionine degradation, K) ubiquitin proteosome system and L) plasma membrane integrity. (JPG 301 KB) [file 441_2022_3678_MOESM6_ESM.jpg]
